# Circularly Polarized Light Emission From Single Chiral Hedgehog Particles Coated with Nanofilms of Achiral Perovskites

**DOI:** 10.1002/adma.202418765

**Published:** 2025-09-04

**Authors:** Michael Veksler, Jeffery Raymond, Tao Ma, Nadine Schrenker, David Fairhurst, Ravi Sharma, Sara Bals, Nicholas A. Kotov

**Affiliations:** ^1^ Department of Chemical Engineering University of Michigan Ann Arbor MI 48109 USA; ^2^ Center of Complex Particle Systems (COMPASS) University of Michigan Ann Arbor MI 48109 USA; ^3^ Biointerfaces Institute University of Michigan Ann Arbor MI 48109 USA; ^4^ Michigan Center for Materials Characterization University of Michigan Ann Arbor MI 48109 USA; ^5^ EMAT University of Antwerp Groenenborgerlaan 171 Antwerp B‐2020 Belgium; ^6^ Mageleka, Inc. 3122 Heather Court Naples FL 34114 USA; ^7^ Department of Materials Science and Engineering University of Michigan Ann Arbor MI 48109 USA

**Keywords:** circularly polarized luminescence, complex particles, differential scattering, flower‐like particles, hedgehog particles, nanostructured microparticles

## Abstract

Metal‐halide perovskites are known for their strong and tunable luminescence. However, the synthesis of perovskite‐based particles with circularly polarized light emission (CPLE) remains challenging due to the complex interplay of metal‐ligand chemistries, crystallization patterns, and chirality transfer mechanisms. Achiral perovskites can be deposited on chiral “hedgehog” particles (CHIPs) with twisted spikes, producing chiroptically active materials with spectroscopic bands specific to the perovskite and chirality specific to the template CHIPs. Left‐ and right‐handed CPLE is engineered into complex particles comprised of a layer of perovskite deposited onto CHIPs coated with an intermediate silica layer. The spectral position of chiroptical bands, the optical asymmetry *g*‐factors, and single‐particle circularly polarized microscopy indicate that the observed CPLE is dominated by the post‐emission scattering from the twisted spikes of the parent particle. Templating luminescent nanofilms on CHIPs provides a simple pathway to a wide range of complex chiroptical materials; the dispersibility of the CHIPs in various solvents and the tunability of their chiral geometry enable their applications as single‐particle emitters with strong and controllable polarization rotation.

## Graphical abstract

Left‐ and right‐handed circularly polarized light emission (CPLE) is observed from chiral “hedgehog” particles (CHIPs) coated with a nanofilm of achiral perovskite, with spectroscopic bands specific to the perovskites and chirality specific to the template CHIPs. Careful analysis of optical activity indicates that the CPLE of perovskite luminescence is dominated by post‐emission scattering from the chiral surfaces of the same CHIPs.

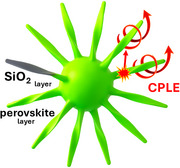

## 1 Introduction

Chiral nanoparticles (NPs) and their assemblies, inclusive of nanostructured microparticles, display multiple scales of chirality, from angstrom to micrometer scale.^[^ ^1^ ^]^ The high polarizability and multiscale chirality of inorganic nanostructures also lead to giant ellipticity across the entire electromagnetic spectrum from ultraviolet to visible, infrared, and terahertz (THz) wavelengths,^[^ ^2^ ^]^ which differentiates them from chiral molecules. These intrinsic properties of chiral nanostructures make them a valuable materials platform for biomedical applications,^[^ ^3^, ^4^, ^5^ ^]^ sensing,^[^ ^6^, ^7^, ^8^ ^]^ catalysis,^[^ ^9^, ^10^ ^]^ and non‐linear optics.^[^ ^11^, ^12^, ^13^ ^]^ An exciting emerging field in chiral nanostructures is particles with circularly polarized light emission (CPLE), making possible their implementation in information technologies,^[^ ^14^ ^]^ optoelectronic devices,^[^ ^15^, ^16^, ^17^ ^]^ quantum computing,^[^ ^18^, ^19^, ^20^ ^]^ memory storage,^[^ ^21^ ^]^ and spintronics,^[^ ^22^, ^23^, ^24^ ^]^ especially when CPLE can be consistently imparted to and encoded into single particles. Note that CPLE encompasses all sources of polarization rotation in the light emitted by the material, as opposed to circularly polarized luminescence (CPL) that refers, strictly speaking, only to the circularly polarized photons emitted as a result of electron transition from excited to ground states, i.e. luminescence.

The common approach to the synthesis of chiral NPs combining luminescence and chirality is the transfer of mirror‐asymmetric surface ligands to light‐emitting semiconductor cores.^[^ ^1^, ^25^, ^26^, ^27^, ^28^ ^]^ However, this synthetic approach does not guarantee circularly polarized luminescence or other optical activity of the NPs beyond the circular dichroism of the ligands.^[^ ^29^, ^30^, ^31^, ^32^ ^]^ Typically, these synthetic protocols also introduce surface trap states and chiral (twist) distortions of the semiconductor crystal lattice, reducing the quantum yield of the nanomaterial.^[^ ^26^ ^]^ Furthermore, finding synthetic pathways to mirror‐asymmetric nanostructures with CPLE is often experimentally tedious due to the non‐stoichiometry of composition of NPs. The multiplicity and variability of non‐classical crystallization pathways of NPs, including their self‐assembly, that are strongly affected by chiral and achiral impurities, also contribute to the synthetic perplexity. The induced chiroptical effects are, furthermore, extremely sensitive to metal‐ligand chemistries because chiroptical activity is strongly dependent on the electric and magnetic moments of the electron‐hole recombination determined by slight variations in the dihedral angles and overlap of atomic orbitals.

Controlled assemblies of NPs, leveraging the chirality of the force‐fields around them, offer much stronger light‐matter interaction with circularly polarized light due to the better dimensional matching compared to small ligand‐stabilized NPs and thus stronger CPL and CPLE.^[^ ^33^ ^]^ They enable the direct transfer of the chirality of photons to matter^[^ ^34^, ^35^ ^]^ and rapid 3D printing on a variety of surfaces.^[^ ^36^ ^]^ However, the growth of chiral nanostructures using this method is specific, at least at this stage of development, to plasmonic materials that do not possess strong luminescence.

Chiral metal halide perovskites attract attention due to their strong light emission paired with the ease of inducing optical activity either through surface modification with chiral ligands or incorporation of small chiral molecules into the crystal lattice.^[^ ^27^, ^37^, ^38^ ^]^ However, similarly to NPs, the degree of ellipticity of the emitted light and the quantum yield of light emission are contradicting properties. Besides polarization rotation of the luminescent photon, the distortions of perovskite octahedra engendering polarization rotation also result in a significant increase in nonradiative recombination.^[^ ^27^, ^39^, ^40^ ^]^ Higher energy of the distorted lattice exacerbates the sensitivity of the crystallization patterns to reaction conditions.^[^ ^41^ ^]^ Hybrid organic‐inorganic perovskites (HOIPs) possess near‐unity photoluminescent quantum yields (PLQY), with LEDs based on HOIPs reaching external quantum efficiencies exceeding 20%.^[^ ^42^, ^43^, ^44^ ^]^ Yet, light emission from chiral HOIPs has mostly been restricted to cryogenic conditions^[^ ^40^, ^45^ ^]^ with ellipticity typically diminishing sharply as temperature increases^[^ ^45^, ^46^ ^]^ despite some promising exceptions utilizing approaches such as energy funneling^[^ ^47^, ^48^ ^]^ and spin injection.^[^ ^49^, ^50^ ^]^

Addressing these challenges, here we explore a pathway to chiral nanostructures that can mitigate the contradictory relationship between fluorescence ellipticity and its quantum yield, building upon the idea of chiral templates.^[^ ^51^, ^52^, ^53^, ^54^ ^]^ Unlike previous methods using nanostructures^[^ ^55^, ^56^, ^57^ ^]^ or liquid crystals,^[^ ^58^, ^59^, ^60^, ^61^, ^62^, ^63^, ^64^, ^65^ ^]^ we employ chiral “hedgehog” particles (CHIPs) with twisted spikes.^[^ ^66^ ^]^ These hierarchically organized particles colloids were previously described as coccolith‐like particles (CLIPs) in the context of de novo synthesis of highly complex structures mimicking skeletons of algae. Their description as CHIPs is used here because it is more more general. There are a large number of possible particles with chiral spikes and the observed optical phenomena are expected to be applicable to them as well. Specifically, we deposit coatings (referred to here also as nanofilms) of highly luminescent formamidinium lead halide (FAPbX_3_) onto CHIPs that also have their own strong red‐orange light emission. Although the composition of FAPbX_3_ is achiral, it results in strong CPLE in the optical bands of perovskite. Circular polarization of the perovskite emission can be changed from left‐ to right‐handed by varying the helicity of the spikes. The spectral position and chiral polarization of CPLE can be varied with minimal modification of the deposition protocol or crystallization parameters, enabling rapid adaptation to application requirements and broad compatibility with other chiral scaffolds and achiral luminescent materials. Multifaceted analysis of the emission spectra and single‐particle CPLE microscopy images indicate that elipticity of the perovskite's emission should be attributed to the post‐emission scattering of photons by the twisted spikes of the same CHIP particle they originated from.

## 2 Results and Discussion

### 2.1 Synthesis and Characterization

**Figure 1:**
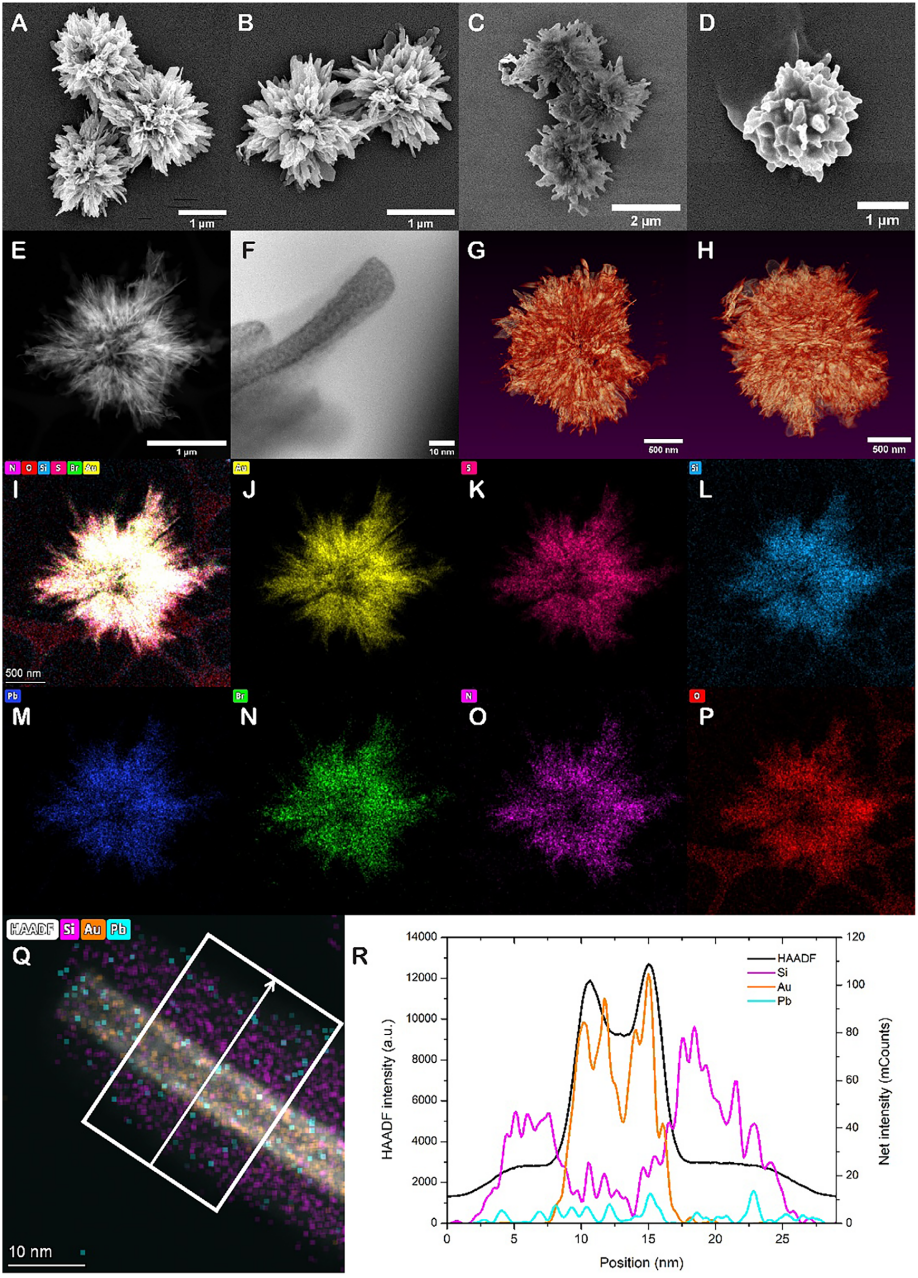
SEM images of a) *L‐*Cys and b) *D‐*Cys CHIPs as‐synthesized at 30 °C. SEM image of *L‐*Cys 30 °C c) S‐CHIPs and d) P‐CHIPs. e) STEM‐HAADF image of a P‐CHIP. f) BF‐STEM image of a spike of a P‐CHIP. This image shows the SiO_2_ coating to be retained after perovskite coating, and ≈10nm thick. g,h) Reconstructed STEM tomography of a P‐CHIP. i–p) Chemical element mapping of selected constituent elements of FAPbBr_3_‐coated P‐CHIP. The imaging results are consistent with CHIPs initially coated with SiO_2_, and subsequently coated with lead bromide perovskite. q,r) EDS line profile on the tip of a FAPbBr_3_‐coated P‐CHIP spike. We note that perovskite films are notoriously sensitive to electron bean and are easily destroyed during imaging. The EDS signatures of lead atoms uniformly distributed over the entire surface indicate the uniformity of the perovskite coating.

CHIPs were synthesized via the electrostatically restricted self‐assembly of gold sulfide nanosheets carrying *L‐* or *D‐*cysteine (Cys).^[^ ^66^ ^]^ For brevity in the enantiomer notations, the corresponding particles will be referred to hereafter as *L‐*Cys and *D‐*Cys CHIPs (**Figure** **1**a,b; Figure S4a–b, Supporting Information). As‐synthesized CHIPs display a gradually increasing overall diameter from 1 to 10 µm as the synthesis temperature increases from 20 to 70 °C. To produce surface‐modified CHIPs carrying nanofilms of perovskites, we took advantage of enhanced dispersibility of the hedgehog particles in aqueous and organic solvents.^[^ ^67^ ^]^ The enhanced dispersibility and slow sedimentation rate of spiky particles in a variety of solvents are related to the drastic reduction of van der Waals attraction between the spiky particles that leads to dominant effective electrostatic repulsion. Since the surface area of adsorbing ions on the tightly bound Stern layer is also increased, the repulsive interactions are also enhanced when the surface is spiky.^[^ ^67^ ^]^

Hydrolysis of TEOS^[^ ^68^ ^]^ in the presence of CHIPs dispersed in ethanol results in a uniform layer of SiO_2_ on them; the corresponding particles with be referred to as S‐CHIPs (Figure 1c; Figure S5a, Supporting Information). The SiO_2_ coating has an average thickness of ≈10 nm (Figure 1f). The thin SiO_2_ layer preserves the complex nano, meso‐, and microscale geometry of these unusual particles. We note that the spiky morphology of CHIPs reduces their aggregation in unfriendly solvents, which is essential for (1) preparation of the silica and perovskite nanofilms and (2) understanding their photophysics. Using ligand‐assisted antisolvent precipitation,^[^ ^69^ ^]^ a layer of polycrystalline perovskite is deposited on the S‐CHIPs rather than on the original “hedgehogs” because silica‐coated S‐CHIPs display greater chemical robustness and compatibility with perovskites than “bare” gold‐thiolate nanostructures. The target perovskite‐coated chiral hedgehog particles (P‐CHIPs) are formed by dispersing S‐CHIPs in an N,N‐DMF solution containing PbX_2_ and FAX precursors, along with oleic acid and oleylamine (Figure S1, Supporting Information). The particle suspension is then directly injected into chloroform, serving as an antisolvent at room temperature, resulting in a layer of perovskite (Figure 1d; Figure S5b, Supporting Information). The perovskite coatings reduced particle roughness and the aspect ratio of the spikes. Scanning transmission electron microscopy (STEM) of P‐CHIPs indicates that the silica layer is smooth and conformal (Figure 1e,f). The same can be inferred about the perovskite coating from the SEM images.

Further analysis of the 3D structure was accomplished by STEM tomography (Figure 1g,h; Movie S1, Supporting Information). This technique confirmed that the morphology of P‐CHIPs preserved the complex hierarchical organization of the spiky CHIP precursors. EDS mapping of P‐CHIPs as a whole (Figure 1i–p) indicates the expected elemental composition containing well‐defined strata of gold sulfide, silicon dioxide, and lead halide. Although it was not possible to determine the thickness of the perovskite layer from electron tomography or 4D STEM data due to the ease of beam damage, an EDS line profile was conducted on the end of one of the P‐CHIP spikes (Figure 1q–r), which shows a core ≈8 nm gold sulfide ribbon surrounded by a layer of ≈10 nm silica and a nano film of perovskite. While the HOIP mainly appears to be on the surface of the silica and TEM does not suggest any porosity of the silica layer, we do not discount the possibility of the perovskite intruding into the silica coating due to its nanoscale porosity.^[^ ^70^ ^]^

Solvent relaxation NMR spectroscopy was conducted on colloidal suspensions of CHIPs, S‐CHIPs, and P‐CHIPs in various solvents (Figure S2 and Table S4, Supporting Information) to better understand the material‐liquid interface and to confirm changes from deposition of SiO_2_ and perovskite layers on the ensemble of particles as opposed to individual CHIPs, as in electron microscopy. A decrease in solvent relaxation time is indicative of an increase in surface wettability.^[^ ^71^ ^]^ The coating of hydrophilic silica onto the CHIPs decreased solvent relaxation times in H_2_O (1701 ms CHIPs vs 164.8 ms S‐CHIPs), as well as in non‐polar solvents such as toluene (2525 ms CHIPs vs 1555 ms S‐CHIPs), signifying increased wettability in a variety of solvents. The coating of perovskite onto S‐CHIPs resulted in equal dispersibility in ethyl acetate (726 ms S‐CHIPs vs 722 ms P‐CHIPs) and only slightly poorer dispersibility in toluene (1555 ms S‐CHIPs vs 1878 ms P‐CHIPs). These findings convincingly indicate omnidispersibility of the spiky nanocolloids and improved wettability of the ligand‐stabilized perovskite surface of P‐CHIPs in non‐polar solvents compared to the original CHIPs.

### 2.2 Photophysical Properties

Deposition of the perovskite layer results in bright photoemission complementing the native luminescence of CHIPs. The improvements in the luminescence intensity can be illustrated by the ten‐fold increase in the PLQY that reaches 3% in the unmodified CHIPs^[^ ^66^ ^]^ compared to the PLQY of 31.3% in *L*‐Cys 30 °C P‐CHIPs coated with FAPbBr_3_. In comparison, the PLQY of similarly synthesized FAPbBr_3_ NPs was reported to be 84%.^[^ ^69^ ^]^ While the P‐CHIPs are bright, their PLQY maxima are smaller than those of pure perovskite NP dispersions. Several phenomena may contribute to quantum yield reduction: i) emission scattering off of parent or neighboring particles may enhance reabsorption and other extinction processes, ii) interfacial effects at the perovskite‐silica interface may reduce the oscilator strength of the radiative recombination from the excited state, and iii) formation of trapped surface states may increase non‐radiative recombination. The presence of the latter effect in P‐CHIPs is substantiated by the elongated emission lifetimes.

**Figure 2:**
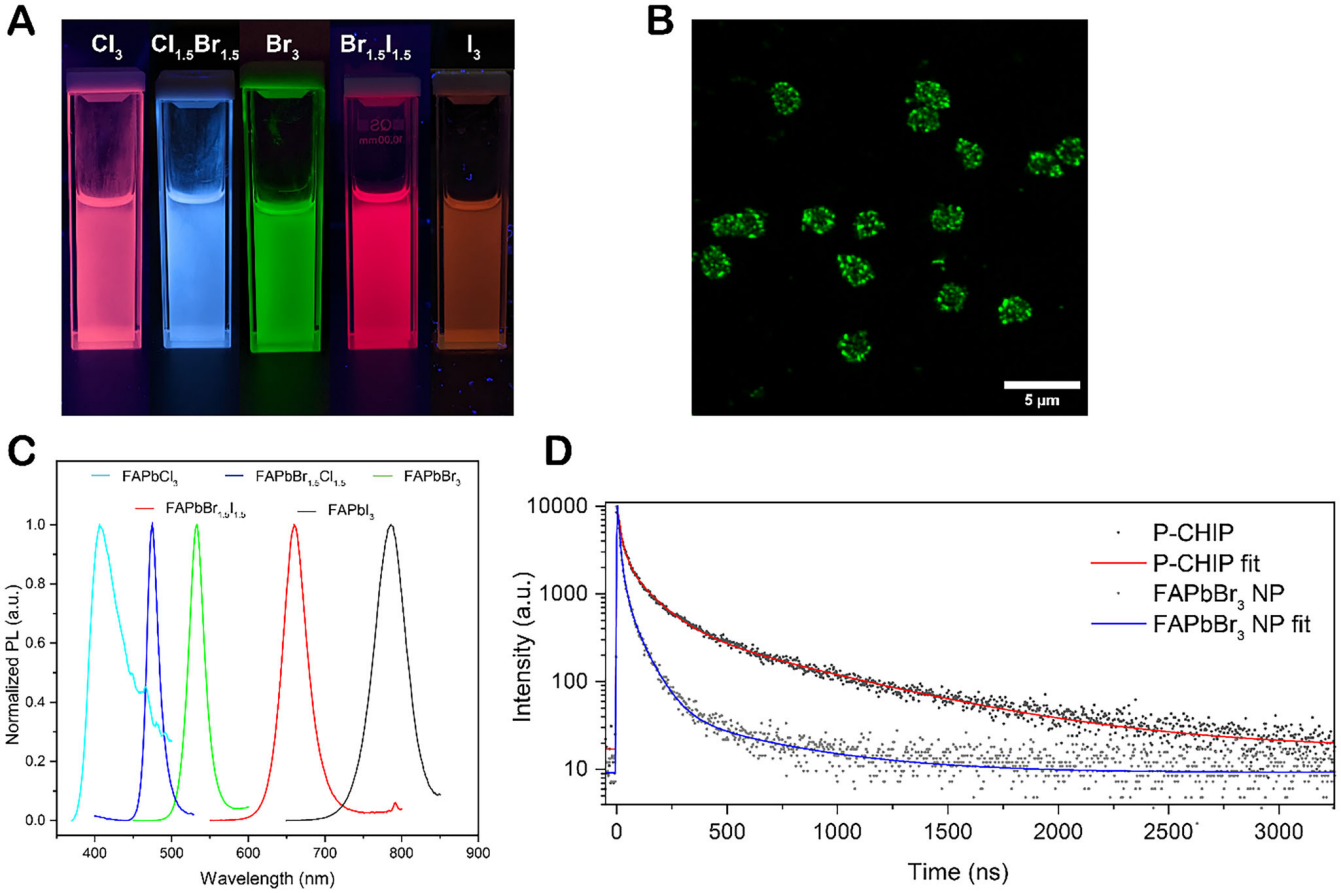
Optical properties of P‐CHIPs. a) Photograph of cuvettes containing colloidal dispersions of FAPbX_3_ P‐CHIPs in toluene under UV irradiation. In the case of FAPbCl_3_‐coated P‐CHIPs, the native red fluorescence of the CHIP template overpowers the light blue fluorescence of the perovskite coating. b) Z‐stack maximum intensity projection of confocal fluorescence micrograph (535 nm emission, 565 nm excitation) of FAPbBr_3_‐coated 30 °C P‐CHIPs (image enhanced with Leica LIGHTNING feature). The microscope utilizes a white light laser and chromatic bean splitters on excitation/emission sides. Note that the SiO_2_ layer is transparent for these illumination conditions. c) Normalized fluorescence spectra of FAPbX_3_ P‐CHIPs (native CHIP fluorescence omitted where measurable). d) Emission decay kinetics for FAPbBr_3_ P‐CHIPs and lab‐synthesized control FAPbBr_3_ NPs in toluene (registered 540 nm emission, 390 nm excitation).

The spectral position of the new band can be tuned from blue to green to infrared as the halide composition of the perovskite precursor changes from Cl to Br to I (**Figure** **2**a). Standard confocal microscopy of P‐CHIPs coated with FAPbBr_3_ (Figure 2b; Figure S3 and Movie S2, Supporting Information; for circular‐polarization‐resolved confocal microscopy of P‐CHIPs also see below) verifies the electron microscopy data regarding the conformal geometry of the coating (Figure S5b, Supporting Information) and the resulting luminescence of the particles. The bright spots in the images of P‐CHIPs are due to the spiky morphology of the CHIP template, which creates areas of high curvature resulting in light emission “hot‐spots”. P‐CHIPs display narrow fluorescent peaks with a full‐width half‐max ranging from 18 to 54 nm (Figure 2c). The average luminescence lifetime for *L*‐Cys 30 °C P‐CHIPs coated with FAPbBr_3_ is ≈54 ns, which contrasts with a control FAPbBr_3_ NPs with an average lifetime of ≈16 ns (Figure 2d). In comparison, the lifetime was reported to be ≈20 ns in FAPbBr_3_ NPs^[^ ^69^ ^]^ ≈40 ns in FAPBBr_3_ NP thin films,^[^ ^72^ ^]^ and ≈200 ns in polycrystalline FAPbBr_3_ bulk films.^[^ ^73^ ^]^ The FAPbBr_3_ on P‐CHIPs shows both a short‐lived lifetime component similar to the NP solution and longer‐lived components (τ_long_ ≈ 573 ns). The nanoscale confinement of NPs is known to accelerate exciton recombination, but is relaxed in films;^[^ ^72^ ^]^ the polycrystalline nature of the coating on CHIPs creates the likelihood of long‐living surface‐stabilized trapped states.

### 2.3 Chiroptical Activity of P‐CHIPs

The tunability of the light emission spectra from the perovskite coating enables us to examine the light‐matter interactions of complex particles in concert with structural variations. CHIPs of specific size and morphology were judiciously matched with a perovskite composition to display overlapping CD and PL spectra to understand the optical processes contributing to ellipticity of the emitted photons (Table S3, Supporting Information). To highlight the previously mentioned distinction between CPL and CPLE, we will reiterate that the former originates from the emission of quanta due to the chirality of the electronic states involved in it, while the latter is more inclusive. CPLE also encompasses effects after the emission of quanta—polarized or unpolarized—due to their in‐flight interactions with the parent particle, surrounding media, or neighboring particles.^[^ ^32^ ^]^

**Figure 3:**
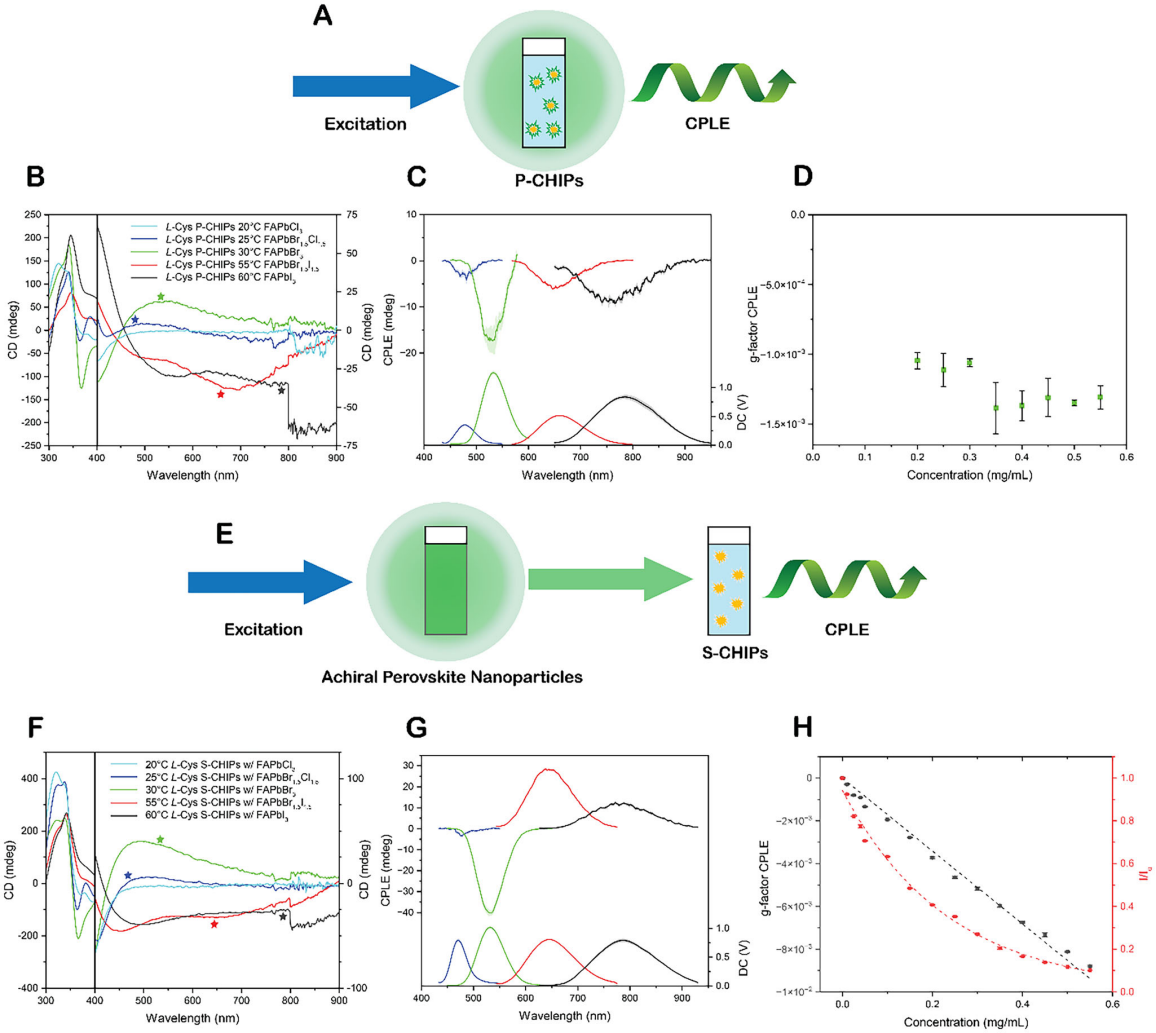
a) Schematic of CPLE measurements of P‐CHIPs in the first configuration (standard in this study). b) CD spectra of *L‐*Cys P‐CHIPs coated with FAPbX_3_ perovskite in toluene. Colors are based on actual fluorescence color, with infrared being black. The spectral position where the fluorescence of the matched perovskite coating corresponds with the CD of the *L‐*Cys P‐CHIPs is marked with a star. c) CPLE of *L‐*Cys P‐CHIPs coated with FAPbX_3_ perovskite. d) *g*_CPLE_ of *L*‐Cys 30 °C FAPbBr_3_ P‐CHIPs at 525 nm across a range of particle concentrations. e) Schematic representing the configuration of fluorescence filtering through dispersed *L‐*Cys S‐CHIPs in CPLE measurement. f) CD spectra of *L‐*Cys S‐CHIPs in toluene. The spectral position where the fluorescence of the matched perovskite NP solution corresponds with the CD peak of the *L‐*Cys S‐CHIPs is marked with a star. g) Resulting CPLE from use of *L‐*Cys S‐CHIPs in toluene as ellipticity‐selective optical filters. h) Resulting *g*_CPLE_ and emission intensity at 530 nm from use of *L‐*Cys 30 °C S‐CHIPs as ellipticity‐selective filters with FAPBBr_3_ NPs across a range of S‐CHIP concentrations.

Specifically, we aimed to determine whether the ellipticity of the luminescence photons originates from emission at the level of individual particles and parent spikes, or if it arises as a collective multiparticle effect through interactions with other CHIPs. Post‐emission polarization of the emitted photons via interactions with the surrounding media and neighboring particles can also be described as circularly polarized ‘internal’ filtering. If internal filtering dominates CPLE generation, the maxima of the CPLE spectra should be directly related to the CD spectra of the P‐CHIPs. Thus, we measured CPLE spectra using the same CHIP‐perovskite pairs in two optical configurations (**Figure** **3**; Figures S6 and S7, Supporting Information). In the first configuration (Figure 3a), CPLE was measured directly from dispersions of P‐CHIPs in toluene. Particles of varying size and perovskite halide composition were used to test the relationship between the peak positions in CPLE and CD. Note that the halide composition of the perovskite layer only slightly affects the CD spectra of the P‐CHIPs as the index of refraction increases from Cl to Br to I (FAPbI_3_ *n* ≈ 2.5).^[^ ^74^, ^75^ ^]^ Also note that there are multiple peaks in the CD spectra exhibiting variable signs, and these peaks broaden and red‐shift as the particles increase in size (Figure 3b; Figure S6a, Supporting Information). In these dispersions, distinct CPLE peaks were observed whose spectral positions correspond nearly perfectly with the fluorescence of the perovskites. For all particles observed here, the sign of the CPLE peaks changes from negative to positive when the twist of the spikes in P‐CHIPs changes from right‐handed in *L‐*Cys P‐CHIPs (Figure 3c) to left‐handed in *D‐*Cys P‐CHIPs (Figure S6b, Supporting Information). The comparison of CD and CPLE characteristics with the size of CHIPs and the synthesis temperature is presented in Table S3 (Supporting Information). Importantly, the sign of the peaks in the CPLE spectra does not always match the sign of the CD spectra at the same wavelength (Figure 3b,c; Figure S6a,b, Supporting Information).

**Figure 4:**
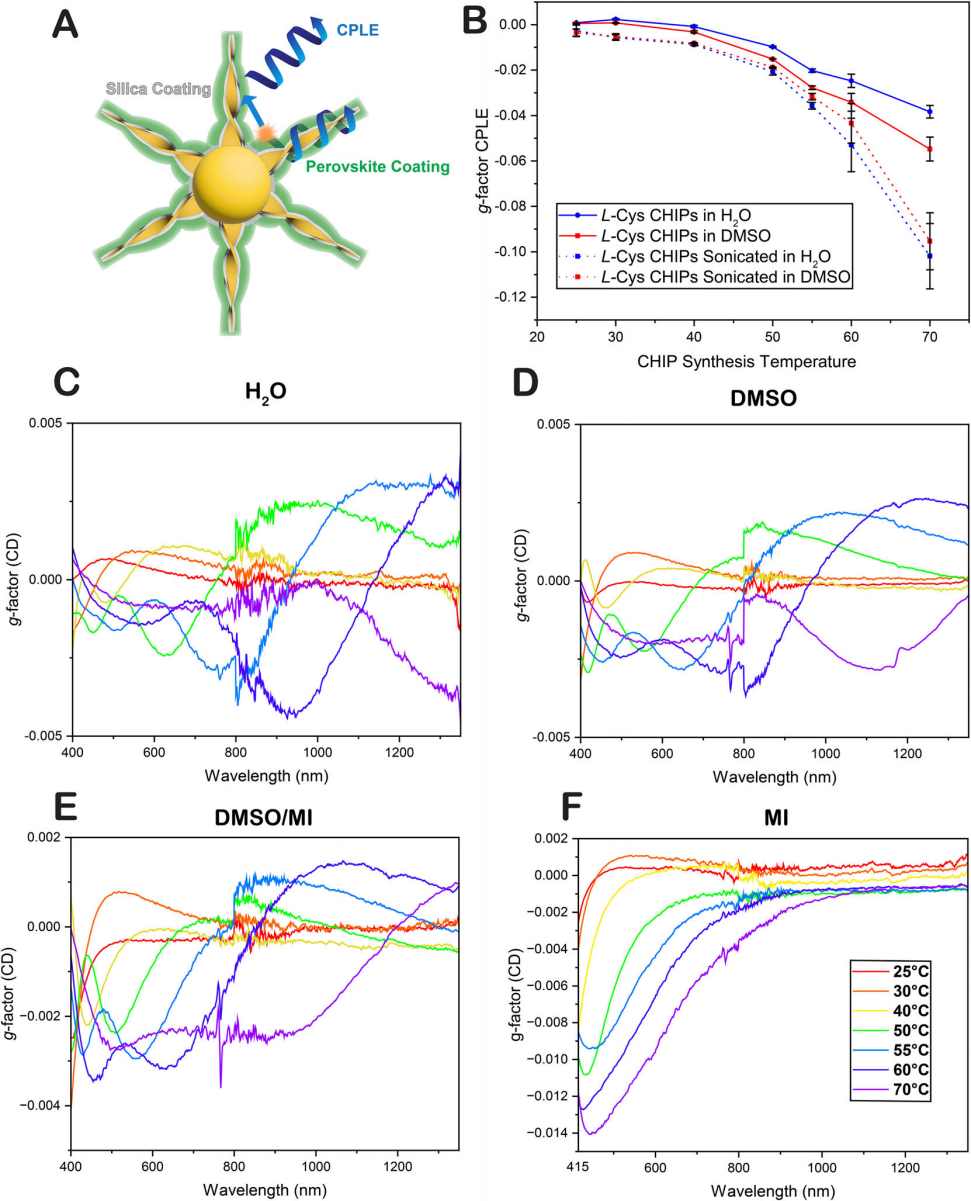
CD and CPLE assessment of CHIPs in solvents of various indices of refraction. a) Schematic illustration of P‐CHIP structure and proposed mechanism of CPLE in P‐CHIPs by scattering. b) *g*‐factor of CPLE from assembled and sonicated *L‐*Cys CHIPs synthesized at various temperatures in water and DMSO. *g*‐factor of CD spectra of *L‐*Cys CHIPs synthesized at various temperatures in c) H_2_O, d) DMSO, e) 50/50% DMSO/MI, and f) MI.

In a second configuration, the dispersions of CHIPs and perovskite NPs are placed in separate cuvettes in a sequence such that the photons emitted by perovskite NPs pass through the dispersion of S‐CHIPs of the same morphologies as those P‐CHIPs measured in the first configuration (Figure 3e). As in the first configuration, the S‐CHIP particles were dispersed in toluene taking advantage again of the omnidispersinility of the hedgehog colloids. The CD spectra of the S‐CHIPs are similar but blue‐shifted in comparison to their P‐CHIP counterparts due to the higher effective refractive index of the P‐CHIPs compared to S‐CHIPs, which red‐shifts and enhances scattering. The S‐CHIPs also exhibit red‐shifted CD spectra as particle sizes increase. In this configuration, the sign of the CPLE peak is consistently opposite to that of the CD spectra of the S‐CHIP at the wavelengths where they overlap with the perovskite fluorescence (Figure 3f,g; Figure S7a,b, Supporting Information), in agreement with prior studies.^[^ ^56^, ^59^, ^64^ ^]^ We conclude that polarization effects due to filtering can predictively determine the sign of the CPLE in this configuration. These data also show that a different mechanism is responsible for the CPLE observed from P‐CHIP dispersions. This ellipticity of the emitted light in P‐CHIPs is associated not with neighboring particles and the internal filtering effect, but with the optical processes intrinsic to the parent particle. Scattering of the emitted photons by the twisted spikes of the parent particle impart experimentally observed ellipticity, (**Figure** **4**a).

Additional evidence comes from CPLE measurements performed on S‐CHIPs and P‐CHIPs in both configurations described in Figure 3a, and 3e across a range of particle concentrations. The CPLE dissymmetry factor, *g*_CPLE_, of FAPbBr_3_‐coated *L*‐Cys 30 °C P‐CHIPs remained nearly constant across varying concentrations (Figure 3d). In contrast, in configuration 2, *g*_CPLE_ increased linearly with S‐CHIP concentration, while the emission intensity decreased exponentially—trends that align with predictions from the Beer‐Lambert law (Figure 3h) (see Supporting Information for additional info). These results again suggest that CPLE from P‐CHIPs originates predominantly from intra‐particle scattering and is not significantly influenced by internal filtering under our experimental conditions.

The importance of minimizing internal filtering when studying native CPLE should be underscored because it can result in misattribution of the optical effects measured in CPL spectrometers. Such conditions can be achieved by reducing particle concentrations and minimizing the optical path length through the chiral medium. Internal filtering in dispersions of chiral particles or other interactions with surrounding chiral media can enhance or suppress CPLE polarization, depending on whether the CD and emission peaks have opposite or similar signs. Careful consideration of internal filtering is especially crucial in CPLE and CPL measurements of films, where optical extinction and scattering are pronounced and known to significantly affect results^[^ ^76^, ^77^ ^]^ in addition to the combined effects of linear dichroism and linear birefringence.

### 2.4 Ellipticity of the Native Luminescence from CHIPs

To attain detailed insights into the origin of circular polarization from P‐CHIPs, we studied their emissive properties further. Specifically, post‐emissive scattering from the parent particle can significantly influence the polarization state of photons emitted from the perovskite or other coatings (**Figure** **4**a).

Original CHIPs without any coatings exhibit red‐orange luminescence at 630 nm related to the electronic states in AuS nanosheets.^[^ ^66^, ^78^ ^]^ Note that typical AuS nanostructures emit non‐circularly polarized luminescence, while in CHIPs, emitted quanta could acquire ellipticity through both luminescence and scattering mechanisms. To understand the origin of CPLE “native” to CHIPs, they were disassembled into constituent twisted ribbons (Figure S4c, Supporting Information) via sonication, which gave us an opportunity to examine how particle assembly contributes to circular polarization effects.

Thus, we focused on how the scattering of CHIPs modulates their intrinsic CPLE (Figure 4b). CPLE measurements were performed on both assembled and sonicated CHIPs (i.e. predominantly nanoribbons) in water (*n* = 1.33) and DMSO (*n* = 1.48); solvents with a higher refractive index, and thus better index‐matched with gold thiolate nanosheets, are expected to reduce scattering cross‐section. CPLE was found to vary considerably with both particle morphology and particle environment (Figure 4b; Figure S8, Supporting Information). Specifically, the *g*_CPLE_ of CHIPs in DMSO was found to lie between that of CHIPs in H_2_O (strong scattering) and that of the sonicated ribbons (weak scattering). This data is consistent with CPLE of CHIPs being modulated by their polarized scattering cross‐section, which both alters the intrinsic CPLE of the red‐emitting AuS CHIPs and contributes to handedness of the photons emitted from the perovskite nanolayer in P‐CHIPs. These results also highlight the significant role of scattering contributions to CPLE in light‐emitting chiral superstructures, an aspect often underexamined.^[^ ^79^ ^]^

Notably, the individual twisted ribbons exhibited stronger CPLE than the assembled particles, and their ellipticity remained nearly unchanged between solvents, consistent with their sub‐wavelength size and minimal scattering. In contrast, the assembled CHIPs demonstrated a strong scattering component, which not only reduced the magnitude of *g*_CPLE_ but also consistently shifted it to a more positive direction. This effect of scattering, resulting in weakening, and positively shifting *g*_CPLE_ across all solvents and morphologies, stood in contrast to the behavior observed in CD spectra (Figures 3f and 4c–f). Interestingly, the dispersive character of the CD spectra of CHIPs, which features oscillating positive and negative bands, appears largely absent from the *g*_CPLE_ spectra.

**Figure 5:**
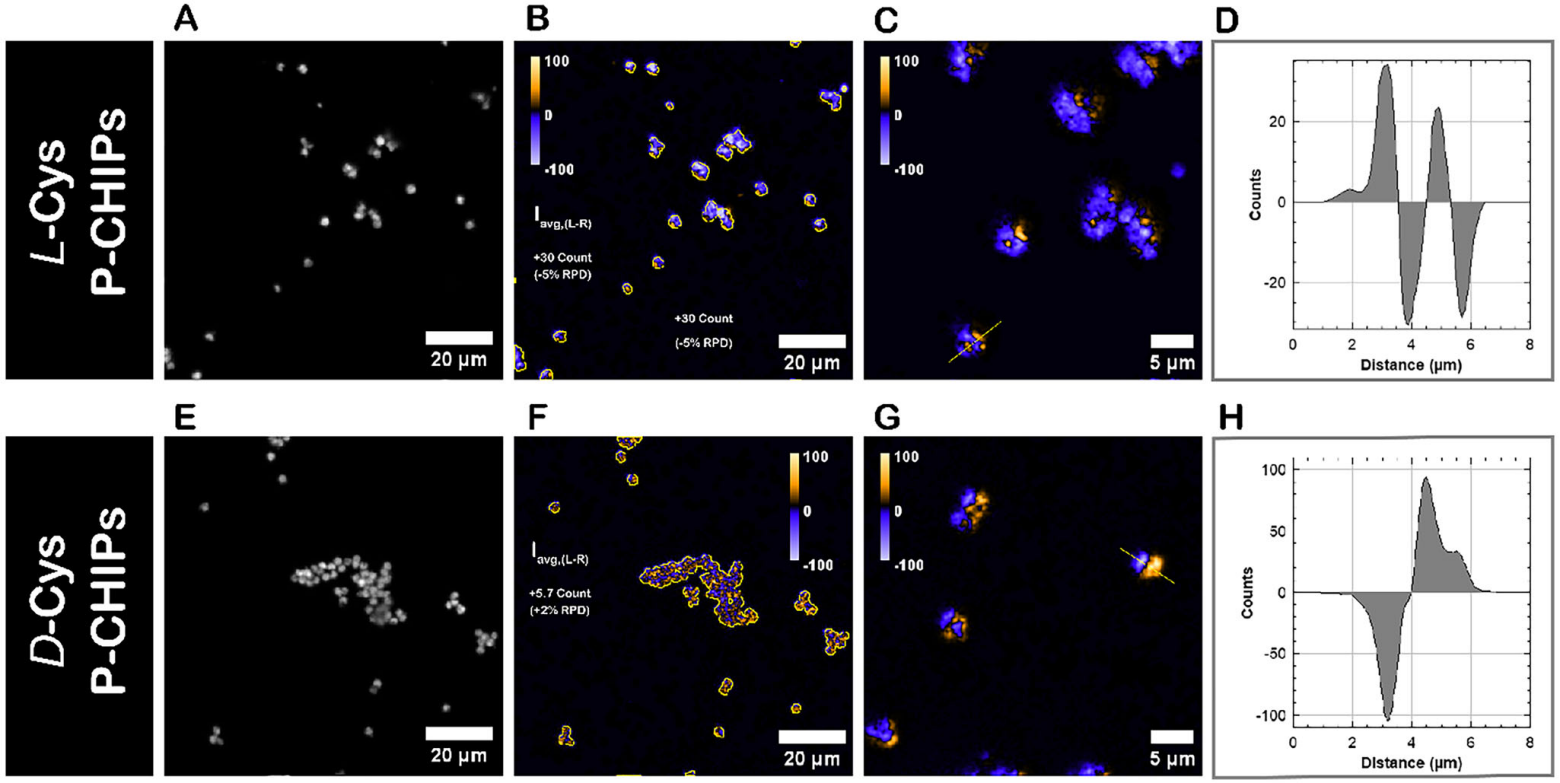
Circularly polarized confocal microscopy (CIRPOM) imaging of single particles for 30 °C FAPbBr_3_ P‐CHIPs of left‐ and right‐handedness. a–d) CIRPOM data for *L‐*Cys P‐CHIPs: a) Average intensity of circularly polarized light emitted by the single hedgehog particles. b) CIRPOM contrast image (relative counts). c) High resolution CIRPOM image. d) Representative line profile intensity for the marked particle in (c). e–h) CIRPOM data for *D‐*Cys P‐CHIPs: e) Average intensity. f) CIRPOM contrast image (relative counts). g) High resolution CIRPOM image. h) Representative line profile intensity for the marked particle in (g).

To further investigate this apparent discrepancy, CD spectroscopy was conducted on *L‐*Cys CHIPs dispersed in solvents and solvent mixtures of progressively higher refractive indices (H_2_O, DMSO, DMSO/MI, and MI). In the highest refractive index medium of methylene iodide (MI) (*n* = 1.74), optical scattering is nearly eliminated; visually, the colloidal suspension appears nearly transparent. The asymmetry *g‐*factors for CD, i.e. *g_CD_*, of the CHIPs dispersed in H_2_O, DMSO, and MI are shown in Figure 4c–f. As the index of refraction increases, the CD bands blue‐shift and reshape as the CHIPs become more closely index‐matched with the solvent and decrease their scattering contribution. Compared with H_2_O, the effective extinction associated with scattering is significantly reduced in MI, while maintaining circular polarization, which results in a *g*_CD_ nearly an order of magnitude larger. The remaining CD intensity is attributed primarily to absorbance rather than scattering mechanisms. The CD amplitude peaked in the blue and diminished toward the red, consistent with index‐matching reducing scattering contributions. Once scattering was suppressed in MI, the strongly dispersive behavior characteristic of CD spectra in other solvents disappeared, indicating that much of the dispersive CD response in mismatched solvents arises from scattering rather than pure absorbance (**Figure** **5**).

Importantly, the CD spectra of CHIPs are not predictive of the ellipticity of circularly polarized scattering, which acts on “native” CPLE. Taken together, these data indicate that the scattering off the parent CHIP's spikes is responsible for modulating native CPLE as it is the case for HOIP nanofilms deposited on CHIPs. We also note that these data for “native” CPLE of all CHIPs cannot be explained by features in their CD spectra. The local optical environment experienced by a fluorophore situated on the surface of the spikes strongly affects its ellipticity. While CD is sensitive to optical extinction, it fails to reveal the directional and polarization‐dependent scattering pathways that modulate photon emission post‐excitation. Thus, the same scattering processes that enhance or suppress CPLE in CHIPs and P‐CHIPs may not have the corresponding peaks in the CD measurements when the light quanta are generated externally. This underscores the need for caution in using CD spectra alone to predict CPLE behavior and highlights the importance of understanding localized scattering effects when interpreting chiroptical activity in complex nanostructures (Figure 4a).

To verify that CPLE generation occurs via scattering off the spikes of parent particles, as depicted in Figure 4a, rather than from neighboring particles, we performed single particle circularly polarized microscopy (which can be abbreviated as CIRPOM) imaging on P‐CHIPs (Figure 5). This custom‐made instrument was developed with the specific purpose of acquiring images of micro‐, meso‐, and nano‐scale particles emitting circularly polarized light (Methods). CIRPOM imaging was conducted on a single z‐slice of 30 °C FAPbBr_3_ P‐CHIPs under left‐ and right‐circular polarization filters, and the difference in left‐ and right‐handed circularly polarized image intensity on a pixel‐by‐pixel basis is presented. We find that on individual particles, both *L‐* and *D‐*Cys, there exist spatial “domains” with an excess of left‐ or right‐handed CPLE. This is consistent with a particle scattering‐dominated process where, atop the complex and corrugated morphology of CHIPs, one would expect large spatial variation of the local scattering environment. Despite this local heterogeneity, the overall handedness of the *single* P‐CHIPs produces a net right‐handed circular polarization intensity in the *L‐*Cys P‐CHIPs, and vice versa in *D‐*Cys P‐CHIPs, with a relative percentage difference (RPD) of −5% in the *L‐*Cys P‐CHIPs and +2% in the *D‐*Cys P‐CHIPs, which is consistent with the ensemble CPLE measurements demonstrated in Figure 3c and Figure S6b in Supporting Information). To rule out polarization bias on the acquiring microscope, results were verified on racemic P‐CHIP particles (Figures S9a and S10, Supporting Information), which show similar domains of excess CPLE that average out to near‐zero (+0.07% RPD) and on non‐chiral FAPbBr_3_ nanocrystals (Figure S9b, Supporting Information), which show evenly unpolarized emission (+0.2% RPD).

While we do not claim full understanding of single‐particle polarization effects and efficiencies, especially given other sources of uncertainty and the limitation of current CIRPOM only being realized on a single z‐slice rather than a summation of full z‐stacks, these results are indicative that individual P‐CHIPs emit CPLE with net excess of circular polarization consistent with the ellipticity observed for dispersions. To confirm, CPLE is not an intrinsic property of the perovskite coating itself, since control perovskite nanofilms are unpolarized. We also want to draw attention to the fact that the observed CPLE is spatially heterogeneous across the particle cross‐section, which was not observed or expected before. Further studies of the differences between the left/right emitting sites would be important.

### 2.5 Spectral and Elliptical Tunability of P‐CHIPs as Single‐Particle Chiral Emitters

**Figure 6:**
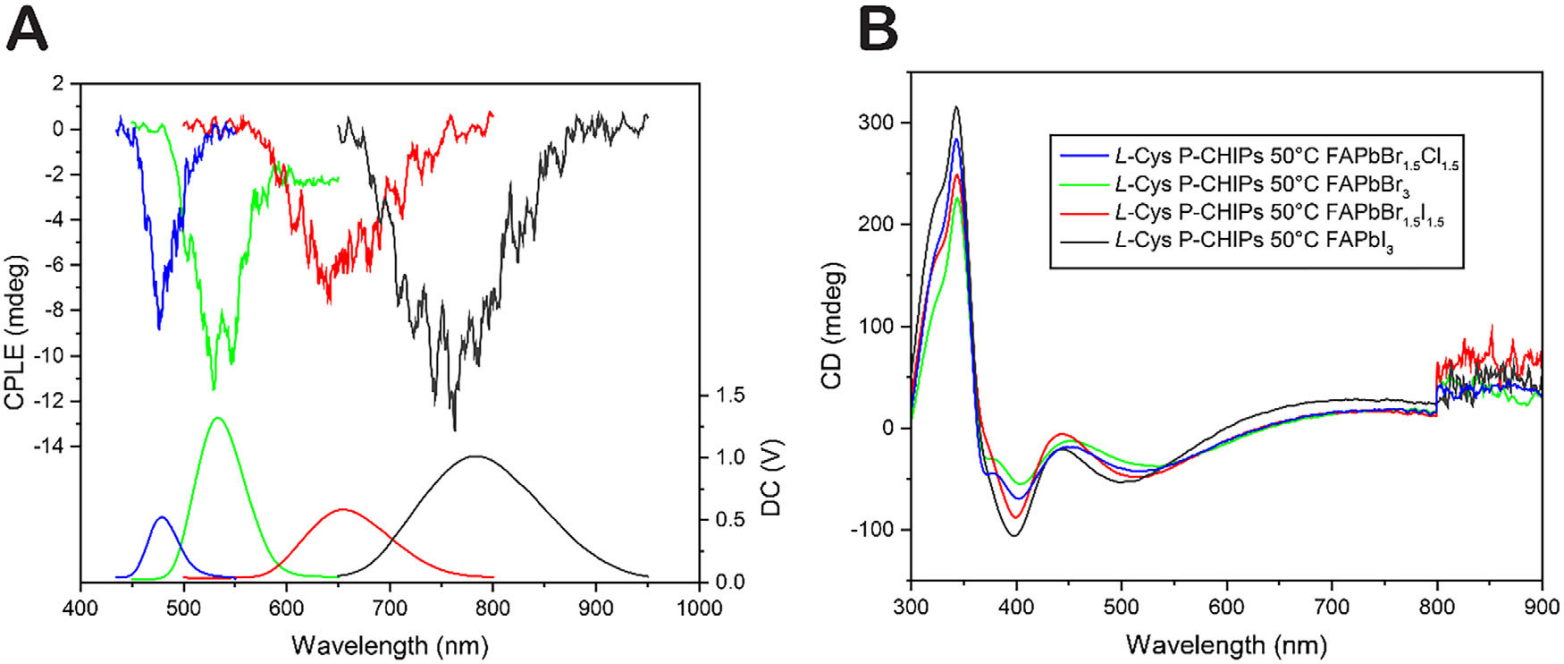
CPLE and CD study of P‐CHIPs coated with nanofilms of perovskite of varied composition. a) CPLE and b) CD spectra of *L‐*Cys 50 °C P‐CHIPs coated FAPbX_3_ with varied perovskite halide composition.

In perovskite‐coated CHIPs, the nanofilms are responsible for emissivity, while the template particles and more precisely their twisted spikes, are responsible for ellipticity. This opens the possibility to resolve the contradictory relationship between intense luminescence and strong polarization rotation prominent in emissive chiral materials. Therefore, we examined the ability of P‐CHIPs to produce CPLE of specific spectral position and polarization, taking advantage of high brightness decoupled from ellipticity. The *L‐*Cys P‐CHIPs synthesized at 50 °C, carrying nanofilms with variable perovskite composition, are shown to produce spectrally tunable CPLE. These particles generate CPLE of similar polarization across the vis‐NIR spectrum simply by modifying the halide composition of the perovskite coating (**Figure** **6**b), which serves as a convenient method to obtain CPLE of desirable color engineered single‐particle sources. As expected from previous data in Figure 3b,c and Figure S6 (Supporting Information), the observed CPLE is not strongly associated with the CD of the particles (Figure 6b), confirming the mechanism in Figure 4a.

**Figure 7:**
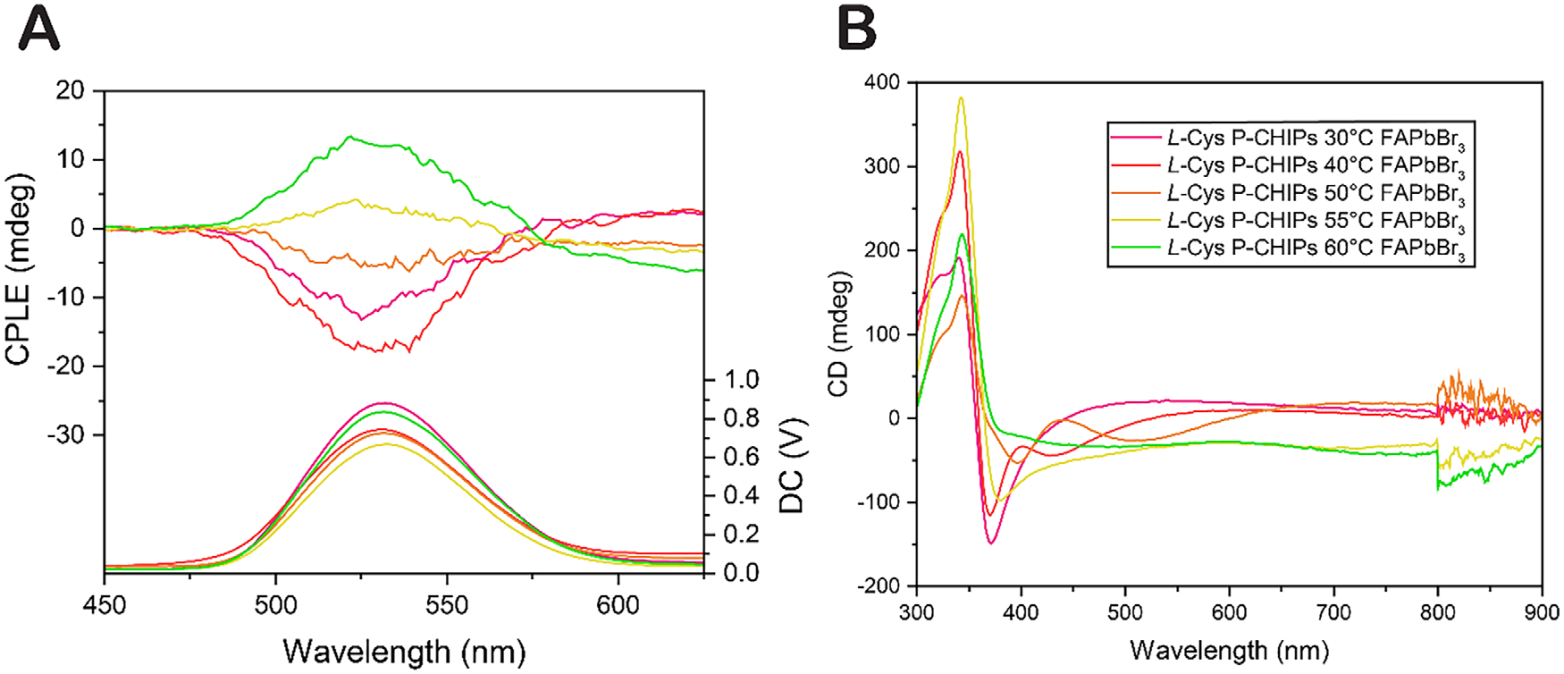
*L‐*Cys P‐CHIPs prepared from CHIPs synthesized at various temperatures, coated with FAPbBr_3_. a) CPLE and b) CD spectra of the dispersions.

To investigate the tunability of ellipticity, FAPbBr_3_‐coated P‐CHIPs were prepared from *L*‐Cys CHIPs synthesized at various temperatures and their CPLE was characterized around a single spectral location (**Figure** **7**). We find that both the sign and magnitude of the polarized emission are strongly correlated with CHIP morphology, which is conventiently and reproducably controlled by the CHIP synthesis temperature. We note that *L*‐Cys P‐CHIPs do not universally provide negative CPLE peaks, exemplified by those *L*‐Cys P‐CHIPs synthesized higher than 50°C in Figure 7 which produce a positive CPLE peak. The singularities of particle geometries and their nano‐, meso‐, and micro‐scale chirality with characteristic dimensions comparable to the wavelength strongly affect the light‐emission patterns.

## 3 Conclusion

This study demonstrates that microparticles with nanostructured twisted spikes, also known as chiral “hedgehogs”, can be coated with nanofilms of highly luminescent perovskites utilizing an intermediate coating of silica. The perovskite‐coated CHIPs exhibit CPLE with remarkable brightness, tunable ellipticity, and spectral location. Additionally, they also have colloidal stability and improved compatibility with non‐aqueous solvents, setting them apart from previous chiral systems with or without perovskites.^[^ ^51^, ^52^, ^53^, ^54^, ^80^, ^81^, ^82^ ^]^

As the central outcome of his work, we conclude that perovskite‐coated CHIPs represent single‐particle emitters where the nanofilms are responsible for emissivity, while the template particles are responsible for ellipticity. The polarization and color of emission are readily tailored by the structure of the particle  and composition of the perovskite nanofilm. Such modularity simplifies the chemical synthesis of emitters of circularly polarized light while uncovering fundamental aspects of light‐matter interactions before and after emission of the quanta.

This approach of decoupling emissivity from chirality 1) opens the door to mitigating the conflicting relationship between photon ellipticity and quantum yield in chiral materials, such as chiral HOIPs; and 2) lays the groundwork for the preparation of other CPLE‐active single‐particle emitters from particles with structural chirality in nanometer and micrometer scales. Such modularity in optical properties makes CHIPs tailorable to the diverse technological requirements of chiral photonics.

## 4 Experimental Section

### Materials

FACl (formamidine hydrochloride, 97%), FABr (formamidinium bromide, ≥98%), FAI (formamidinium iodide), PbCl_2_ (lead(II) Chloride (99.999% Perovskite Bead), PbBr_2_ (lead(ii) bromide ≥99.998%), PbI_2_ (lead(ii) iodide ≥99.9985%), oleic Acid (technical grade, 90%), oleylamine (approximate C18‐content 76.5 – 83.5%), HAuCl_4_∙3H_2_O (Gold(iii) chloride trihydrate, ≥99.9%), L‐cysteine hydrochloride monohydrate (reagent grade, ≥98%), D‐cysteine hydrochloride monohydrate, CTAB (hexadecyltrimethylammonium bromide, BioXtra, ≥99%), ammonium hydroxide solution, PVP (polyvinylpyrrolidone, average mol wt 40000), TEOS (tetraethyl orthosilicate reagent grade 98%), toluene (anhydrous 99.8%), ethanol, and N,N‐dimethylformamide (anhydrous, 99.8%) were purchased from Sigma‐Aldrich. All chemicals were used as received.

### Synthesis of S‐CHIPs

Particles were silica‐coated using a modified procedure from ref. [83]. CHIPs (10 mL, 5 mg mL^−1^) in water were mixed with PVP‐40 (100 mg) overnight. The particles were transferred to ethanol (10 mL) with ammonium hydroxide (0.44 mL). TEOS (60 µL) was added and allowed to stir overnight.

### Synthesis of P‐CHIPs

Formamidinium lead halide perovskite was coated onto S‐CHIPs using a modified procedure from ref. [69]. FaX (0.1 mmol) and PbX_2_ (0.1 mmol) were dissolved in DMF (1 mL, 0.1 m). Then, oleic acid (OA) (200 µL) and X amount (20 µL – 150 µL) of oleylamine (OAm) (refer to Table S1, Supporting Information) were added. S‐CHIPs (2.5 mL, 5 mg mL^−1^) were washed several times with DMF to remove all moisture. They were then drained and mixed with a portion of the perovskite precursor solution containing OAm and OA. Y amount (26–31.2 µL) of DMF (refer to Table S1, Supporting Information) was then added to dilute the perovskite solution (0.05 m). A portion of the solution (80 µL) was then injected into chloroform under stirring. For purification, the particles were centrifuged at 3000 RPM for 3 min to settle the particles and separate the supernatant containing perovskite NPs. The P‐CHIPs were then redispersed in toluene. This process was repeated two more times. Further parameters are provided in Table S1 (Supporting Information).

### Standard Confocal Imaging

Images were acquired on a Leica SP8 confocal microscope. Perovskite‐coated samples were dispersed on a glass slide with silica oil as the slide media. Images were acquired under a 100x magnification lens. A tunable white light laser excitation source was used with excitation at 565 nm. Images were acquired with a single tunable emission channel set to 500–540 nm. Z‐stack images were acquired with 0.3 µL between Z‐slices at 200 Hz speed. Some images were enhanced using the Lightning image processing software with the LAS X software where indicated.

### Thermogravimetric Analysis (TGA)

Particle mass concentrations were determined by dispersing a known aliquot of the colloidal suspension onto a TGA pan and drying at 60 °C until all liquid solvent was removed.

### NMR Solvent Relaxation

The CHIP dispersions were analyzed directly using a *M*agno*M*eter XRS NMR spectrometer, operating at 12.5 MHz, from Mageleka Inc., Naples, FL, USA. A CPMG pulse sequence^[^ ^84^, ^85^ ^]^ was used to measure the spin‐spin relaxation time; a 180° pulse spacing of 1000 µs and up to 20 000 echoes were recorded with a 90° pulse length of 4.5 µs.

### Fluorescence Spectroscopy

Fluorescence spectra were measured on a Horiba Fluoromax Plus using a xenon arc lamp excitation source. Particles with perovskite coating FAPbCl_3_, FAPbCl_1.5_Br_1.5_, FAPbBr_3_, FAPbBr_1.5_I_1.5_, and FAPbI_3_ were excited at 315, 350. 360, 390, and 450 nm, respectively, for fluorescence measurements. The emission spectra were corrected for the spectral responsivity of the fluorometer detector. The absolute fluorescence quantum yield was measured with a calibrated integrating sphere. Fluorescence lifetime measurement (TCSPC) was measured on the same instrument with a 390nm pulsed laser diode.

### Circular Dichroism Spectroscopy

CD spectra were recorded on a JASCO J‐1700 spectrophotometer.

### Circularly Polarized Luminescence Spectroscopy

CPLE spectra were recorded on a JASCO CPL‐300.

### Electron Microscopy

Scanning electron microscopy was acquired on a Thermo Fisher Nova 200 at an accelerating voltage of 5 kV. Samples were sputter‐coated with a thin layer of gold prior to imaging. STEM imaging and EDS elemental analysis were obtained on a Thermo Fisher Talos F200X G2 S/TEM operating at 200 kV. For the STEM and EDS accomplished in conjunction with the EDS line profile shown in Figure 1q,r, STEM images were acquired with a probe‐corrected Thermo Fisher Scientific Titan Microscope operating at 300 kV with a probe semi‐convergence angle of ≈20 mrad, and EDS was performed using a Super‐X detector and a current of 50 pA.

### Tomography

The tomography dataset was acquired by tilting the sample from −70° to 70° with an increment of 2°. The 3D reconstruction was performed with the simultaneous iterations reconstruction technique (SIRT) algorithm using the reconstruction module in Gatan DigitalMicrograph.

### Circularly Polarized Confocal Microscopy (CIRPOM)

A customized confocal‐STED spectral imaging system was utilized to attain left‐handed and right‐handed circularly polarized confocal images. Excitation occurred at 405 nm with pulsed excitation (6 ps FWHM) from a super‐continuum fiber laser operated at 40 MHz and constant flux. A 495 nm long pass dichroic mirror was utilized to exclude the excitation source and associated scattering from the emission signal. A monochromator was utilized to further select an emission wavelength of 530 nm (2 nm slit) prior to a photon counting unit (PMT) that was maintained at identical gain (90%) throughout the study. All pixel dwell times were held constant (1 ms) to ensure comparability between images at different scales. Left‐ and right‐handed CIRPOM images were taken utilizing a broadband (400 to 800 nm) quarter and half‐wave plate pair with fast axis rotation at +45/−45 to the linear polarizer. Images were collected in the ISS *VistaVision* software platform and processed in ImageJ. CIRPOM images were presented based on direct image math on a per pixel basis and shown with a single 1‐pixel gaussian averaging for clarity. A relative percent difference was calculated from an average intensity image after the utilization of thresholding to only consider pixels with sufficient brightness. This region of interest was overlaid onto the images from which the RPD% was calculated. Racemic CHIPs and non‐chiral nanocrystals (see SI for additional information) as controls and demonstrated +0.07% and +0.2% bias, respectively. Collection efficiency was also assessed through excitation scattering collection at 420 nm, and no bias was detected in the excitation source. In all CIRPOM images (left‐handed minus right‐handed) a RH circularly polarized light image was negative, and a LH circularly polarized image was positive.

## Conflict of Interest

The authors declare no conflict of interest.

## Author Contributions

M.V. designed and conducted the experiments. M.V. did the design, synthesis, and characterization of the particles. J.R. conducted CPL confocal imaging and assisted with time‐resolved fluorescence and quantum yield measurements. T.M. assisted with TEM imaging and carried out tomography reconstruction. N.S. and S.B. assisted with TEM imaging and EDS analysis. D.F. and R.S. conducted the NMR solvent relaxation measurements. N.A.K. conceived the project. M.V. and N.A.K. wrote the paper.

## Supporting information



Supporting Information

Supplemental Movie 1

Supplemental Movie 2

## Data Availability

The data that support the findings of this study are available in the supplementary material of this article.
