# Supplementary material for: Circularly Polarized Light Emission From Single Chiral Hedgehog Particles Coated with Nanofilms of Achiral Perovskites
Source: Adv Mater. 2025 Sep 4;37(45):e18765. doi: 10.1002/adma.202418765 (PMC12617027; doi:10.1002/adma.202418765)
Supplement: Supplementary file 1 — Supporting Information [file ADMA-37-e18765-s001.docx]

**Supporting Information**

Circularly Polarized Light Emission from Chiral Hedgehog Particles Coated with Nanofilms of Achiral Perovskites

Michael Veksler, Jeffery Raymond, Tao Ma, Nadine Schrenker, David Fairhurst, Ravi Sharma, Sara Bals, Nicholas A. Kotov^*^

**Table S1.** P-CHIP synthesis parameters adapted from ref. ^[1]^.

| Perovskite Composition | Perovskite Precursor Amount, mL | Oleic Acid, µL | Oleylamine, µL | Precursor added to S-CHIPs, µL | DMF for Dilution, µL | Amount Injected, µL |
| --- | --- | --- | --- | --- | --- | --- |
| FAPbCl_3_ | 1 | 200 | 20 | 48.8 | 31.2 | 80 |
| FAPbCl_1.5_Br_1.5_ | 1 | 200 | 30 | 49.3 | 30.7 | 80 |
| FAPbBr_3_ | 1 | 200 | 40 | 50 | 30 | 80 |
| FAPbBr_1.5_I_1.5_ | 1 | 200 | 95 | 52 | 28 | 80 |
| FAPbI_3_ | 1 | 200 | 150 | 54 | 26 | 80 |

**Table S2.** Photoluminescent Lifetime Tail-Fitted Parameters of *L*-Cys 30°C FAPbBr_3_ P-CHIPs and FAPbBr_3_ NPs (registered 540 nm emission, 390 nm excitation).

|  | a_1_ | τ_1_ | a_2_ | τ_2_ | α_long_ | τ_avg,long_ |
| --- | --- | --- | --- | --- | --- | --- |
| P-CHIPs | 0.45 | 118 | 0.55 | 641 | 20% | 573 |
| NPs | 0.79 | 72 | 0.21 | 477 | 7% | 330 |

**Table S3**. CHIP and P-CHIP pairings and CD/CPLE comparison between configurations 1 and 2 relating to Figure 3.

| Size (Synthesis Temperature) | Perovskite Composition | CD Sign | CPLE Sign | λ_ex_ | λ_em_ |
| --- | --- | --- | --- | --- | --- |
|  | **Configuration 1** |  |  |  |  |
| 1.2 µm (20°C) | FAPbCl_3_ | (-) | N/A | N/A | N/A |
| 1.5 µm (25°C) | FAPbCl_1.5_Br_1.5_ | (+) | (-) | 375 nm | 480 nm |
| 2 µm (30°C) | FAPbBr_3_ | (+) | (-) | 350 nm | 535 nm |
| 5.5 µm (55°C) | FAPbBr_1.5_I_1.5_ | (-) | (-) | 415 nm | 660 nm |
| 6.5 µm (60°C) | FAPbI_3_ | (-) | (-) | 400 nm | 785 nm |
|  | **Configuration 2** |  |  |  |  |
| 1.2 µm (20°C) | FAPbCl_3_ | (+) | N/A | N/A | N/A |
| 1.5 µm (25°C) | FAPbCl_1.5_Br_1.5_ | (+) | (-) | 350 nm | 470 nm |
| 2 µm (30°C) | FAPbBr_3_ | (+) | (-) | 350 nm | 530 nm |
| 5.5 µm (55°C) | FAPbBr_1.5_I_1.5_ | (-) | (+) | 415 nm | 645 nm |
| 6.5 µm (60°C) | FAPbI_3_ | (-) | (+) | 410 nm | 785 nm |


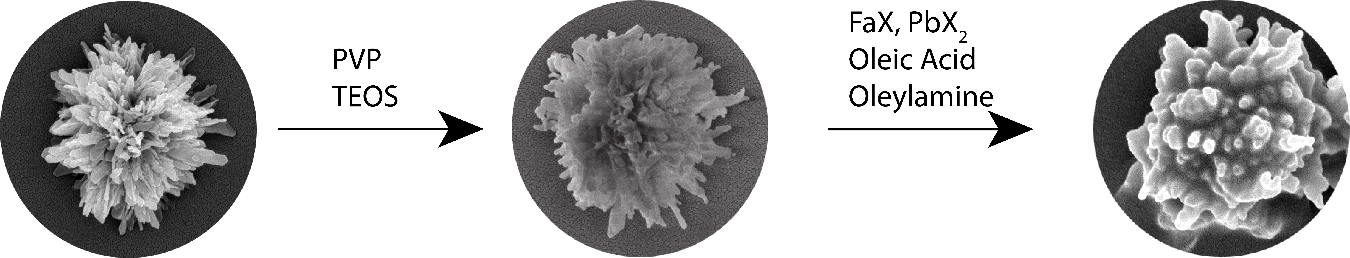


**Figure S1.** Step-by-step diagram of the P-CHIP synthesis.


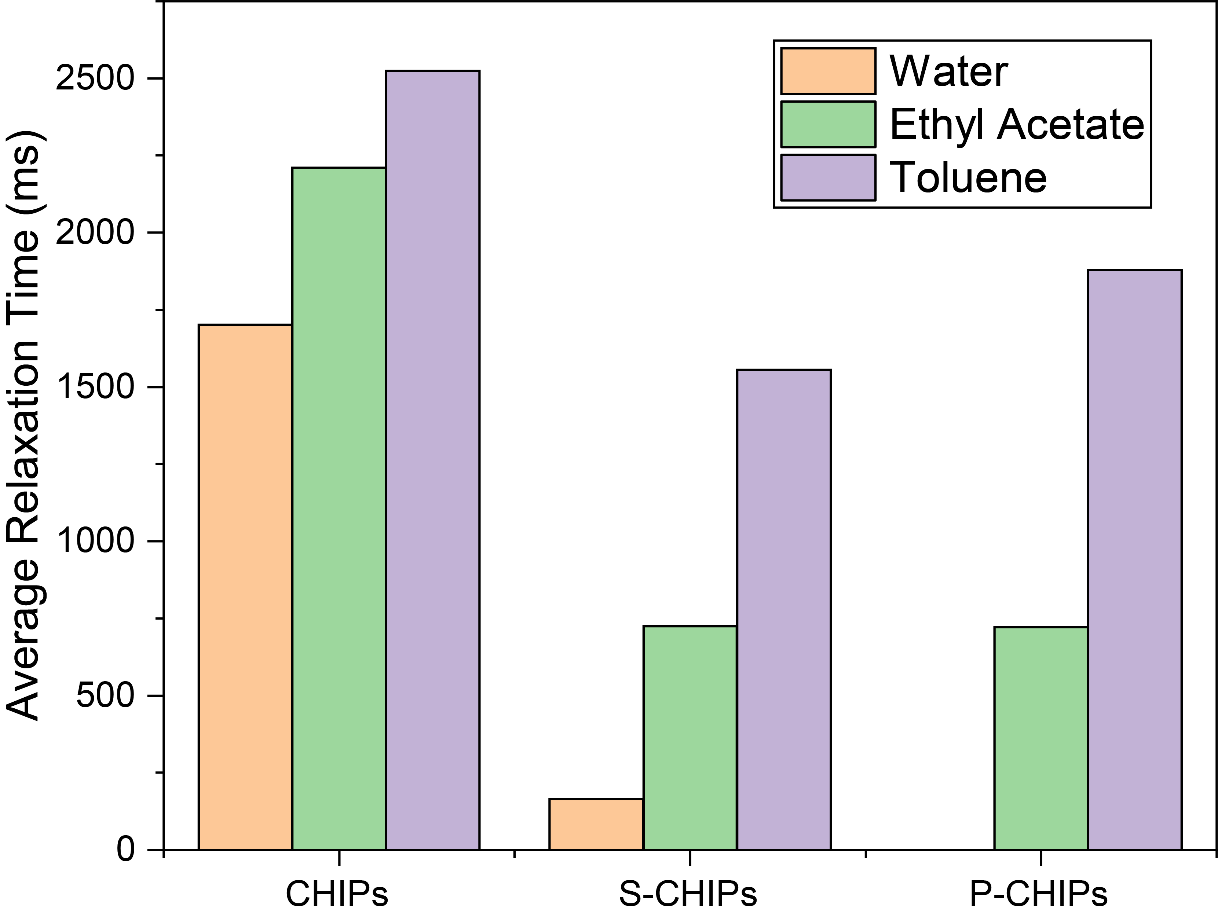


**Figure S2.** Solvent NMR relaxation time of surface-modified CHIPs synthesized at 50°C. Particles were colloidally suspended in water, ethyl acetate, and toluene.

**Table S4.** Solvent NMR relaxation time of surface-modified CHIPs synthesized at 50°C. Particles were colloidally suspended in water, ethyl acetate, and toluene.

|  | **H_2_O** | **Ethyl Acetate** | **Toluene** |
| --- | --- | --- | --- |
| CHIPs | 1701 | 2211 | 2525 |
| S-CHIPs | 164.8 | 725.5 | 1554.9 |
| P-CHIPs | N/A | 722.4 | 1878.4 |

**
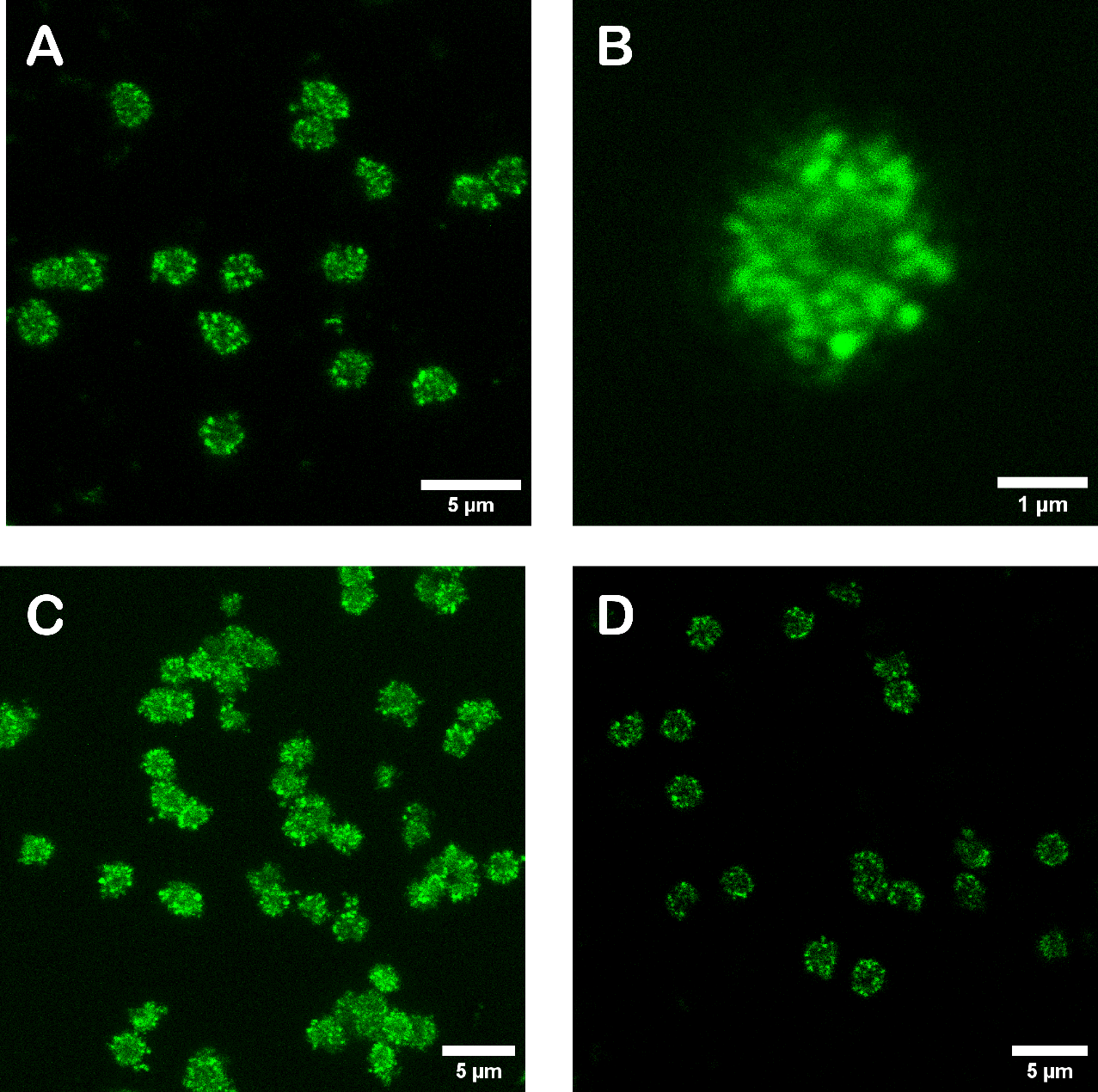
**

**Figure S3.** Standard confocal microscopy images of FAPbBr_3_-coated 30°C *L-*Cys P-CHIPs. (a-c) Max projections of a Z-stack of confocal images with different magnifications. (d) Single Z-slice confocal microscopy image.

**
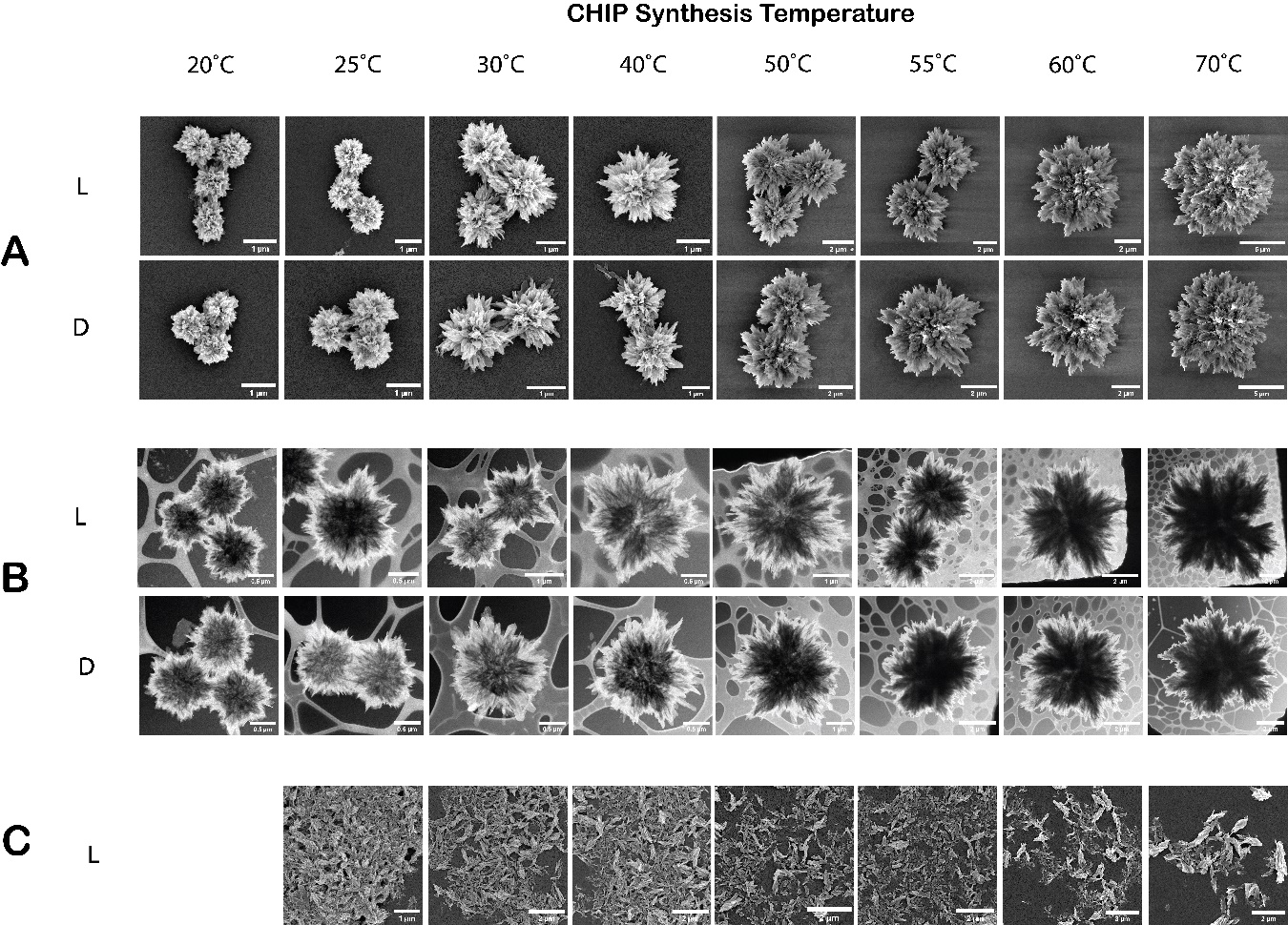
**

**Figure S4.** SEM and TEM images of as-synthesized and sonicated CHIPS. a) SEM and b) DF-STEM images of *L-*Cys and *D-*Cys CHIPs as-synthesized. c) SEM images of *L-*Cys CHIPs after sonication into constituent nanoribbons.


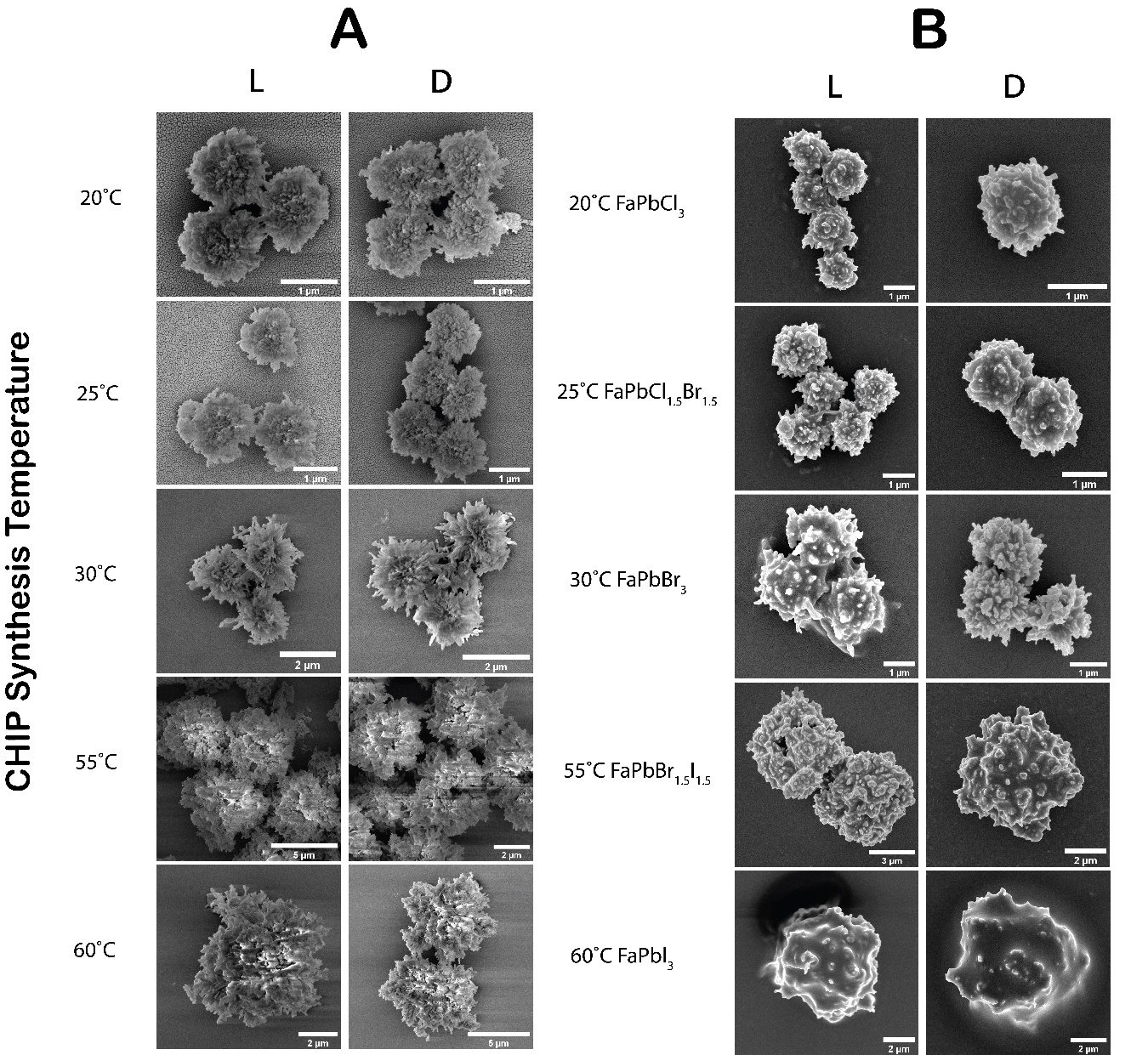


**Figure S5.** SEM images of S-CHIPs and P-CHIPs. a) SEM images of a subset of S-CHIPs. b) SEM images of a subset of P-CHIPs coated with varied perovskite composition.

**
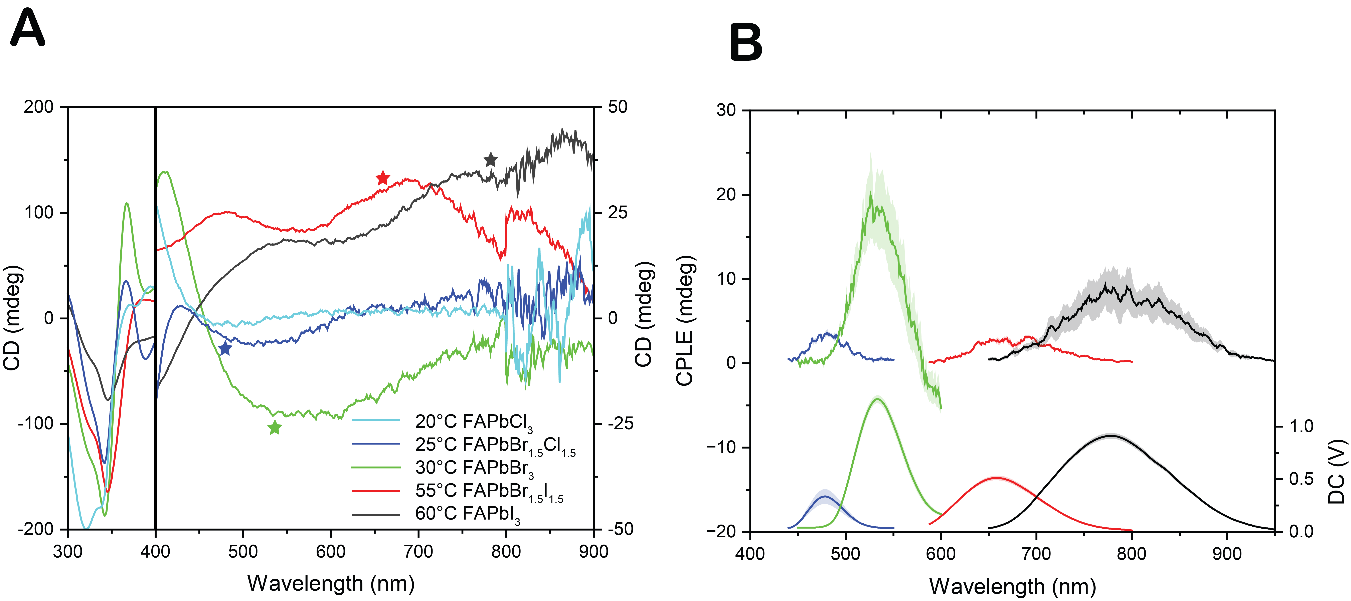
**

**Figure S6.** *D-*Cys P-CHIPs measured in configuration 1. a) CD spectra of *D-*Cys P-CHIPs coated with FAPbX_3_ perovskite. Colors are based on actual fluorescence color with infrared being black. The spectral position where the fluorescence of the matched perovskite coating corresponds with the CD of the *D-*Cys P-CHIPs is marked with a star. b) CPLE of D*-*Cys P-CHIPs coated with FAPbX_3_ perovskite.

**
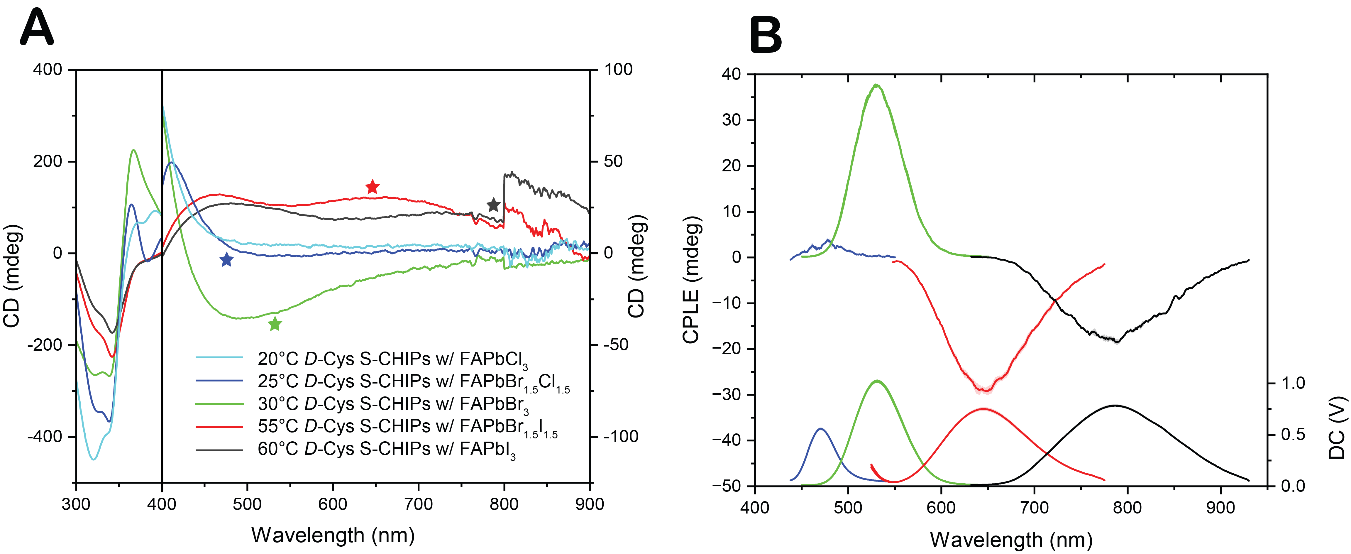
**

**Figure S7.** *D-*Cys S-CHIPs measured in configuration 2. a) CD spectra of *D-*Cys S-CHIPs in toluene. The spectral position where the fluorescence of the matched perovskite nanocrystal solution corresponds with the CD peak of the *D-*Cys S-CHIPs is marked with a star. b) Resulting CPLE from use of *D-*Cys S-CHIPs in toluene as ellipticity-selective optical filters.

**Supplementary Discussion**

In Fig. 3, CPLE is measured in two separate configurations, one in which it is posited that CPLE is not affected by filtering, and a second in which all CPLE is due to polarized filtering. To prove these assertions, we consider the dependency of measured CPLE (in terms of g_CPLE_) on particle concentration.

In configuration 2, the perovskite nanoparticle emission is filtered through a colloidal dispersion of S-CHIPs. The intensity and polarization of the detected light can be described by the Beer-Lambert law. In natural log form:

(1)

$I=I_{0}e^{\varepsilon lc\cdot ln(10)}$

Where $\varepsilon$ is the extinction coefficient due to both absorption and scattering, $l$ is the optical path length of the chiral medium, and $c$ is the concentration of particles. The intensities of both circular polarizations of light are then given by:

(2)

(3)

$$I_{L}=\frac{I_{0}}{2}e^{\varepsilon_{L}lc\cdot ln(10)}$$

$$I_{R}=\frac{I_{0}}{2}e^{\varepsilon_{R}lc\cdot ln(10)}$$

Such that:

(4)

$$\Delta I=I_{L}-I_{R}= \frac{I_{0}}{2}e^{\varepsilon_{L}lc\cdot ln(10)}-\frac{I_{0}}{2}e^{\varepsilon_{R}lc\cdot ln(10)}$$

If we define $\varepsilon$ such that

(5)

$$\varepsilon_{ave}=\frac{\varepsilon_{L}+\varepsilon_{R}}{2}$$

And

(6)

$$\Delta\varepsilon=\varepsilon_{L}-\varepsilon_{R}$$

Then:

(9)

(8)

(7)

$$\Delta I=\frac{I_{0}}{2}e^{{(\varepsilon}_{ave}+\frac{\Delta\varepsilon}{2})lc\cdot ln(10)}-\frac{I_{0}}{2}e^{{(\varepsilon}_{ave}-\frac{\Delta\varepsilon}{2})lc\cdot ln(10)}$$

$$\Delta I=\frac{I_{0}}{2}e^{\varepsilon_{ave}lc\cdot\ln\left( 10 \right)}(e^{\frac{\Delta\varepsilon}{2}lc\cdot\ln\left( 10 \right)}-e^{-\frac{\Delta\varepsilon}{2}lc\cdot\ln\left( 10 \right)})$$

$$g_{CPLE}=\frac{\Delta I}{\frac{1}{2}I}=\frac{\Delta I}{\frac{1}{2}(I_{L}+I_{R})}=2tanh(\frac{\Delta\varepsilon}{2}lc\cdot\ln\left( 10 \right))$$

At small values of x, tanh(x)~x. Entering this approximation:

(10)

$$g_{CPLE}\approx\Delta\varepsilon lc*\ln\left( 10 \right)$$

Therefore, Beer’s law predicts that the $g_{CPLE}$ increases linearly with concentration, while the average emission intensity undergoes exponential decay. These results are exactly what is observed in Fig. 3h.

On the other hand, in configuration 1 and Fig. 3d, the measured $g_{CPLE}$ from P-CHIPs appears independent of concentration, lending credence that filtering effects are negligible within the experimental parameters used.


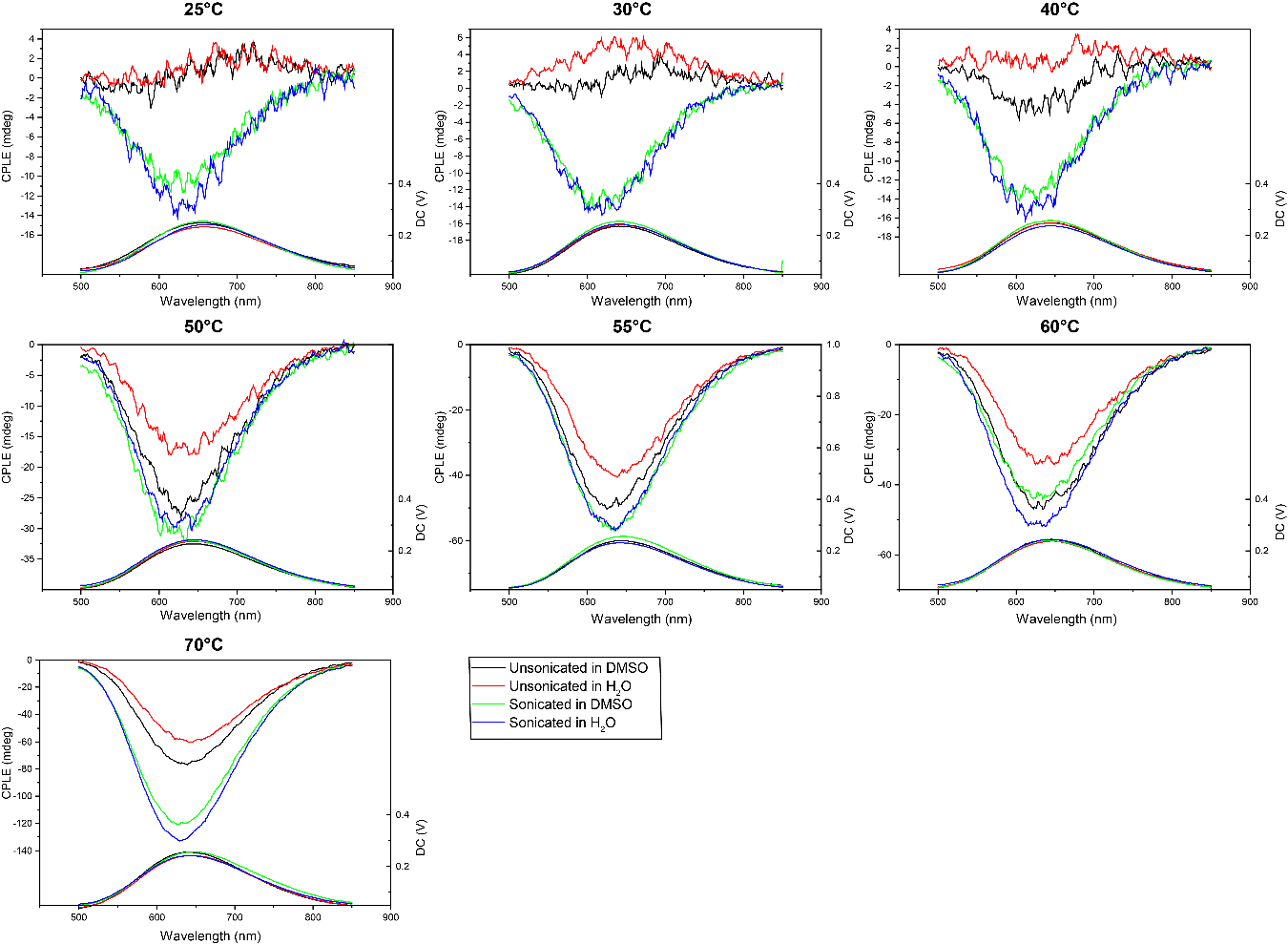


**Figure S8.** Subset of CPLE spectra of *L-*Cys CHIPs measured at variable synthesis temperature, solvent environment, and sonication conditions.

**
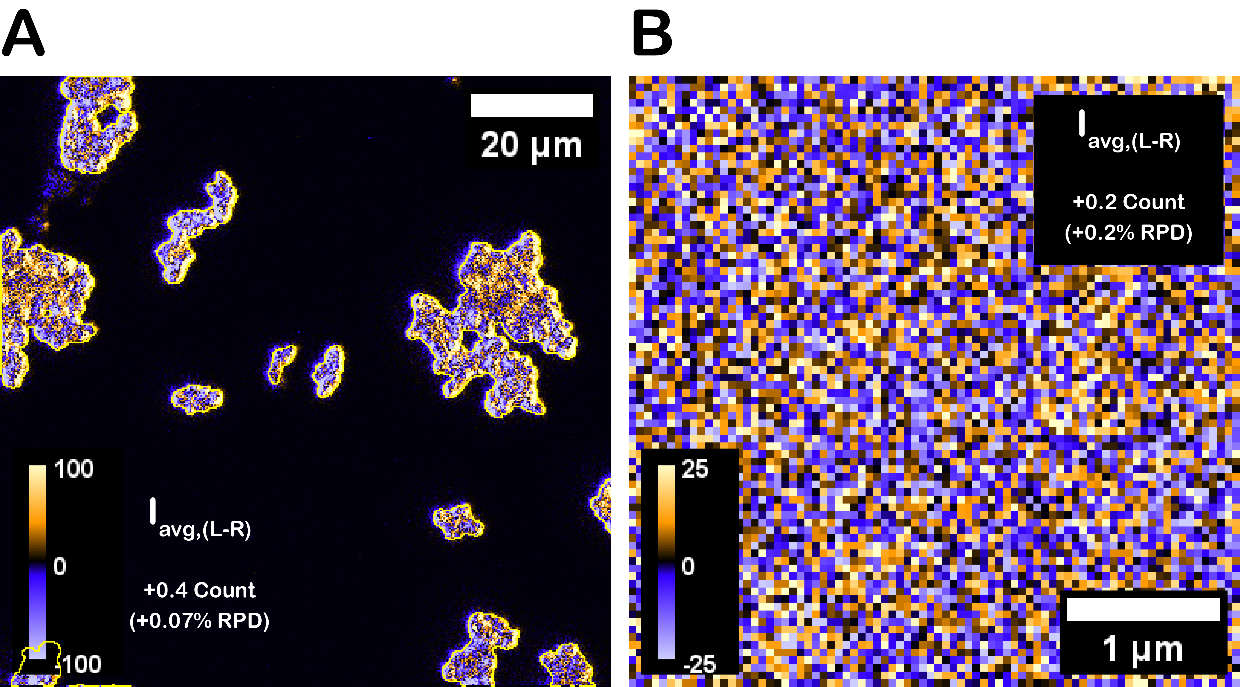
**

**Figure S9.** CIRPOM imaging of a) racemic P-CHIPs coated with FAPbBr_3_ and b) a film of achiral FAPbBr_3_ nanocrystals.

**
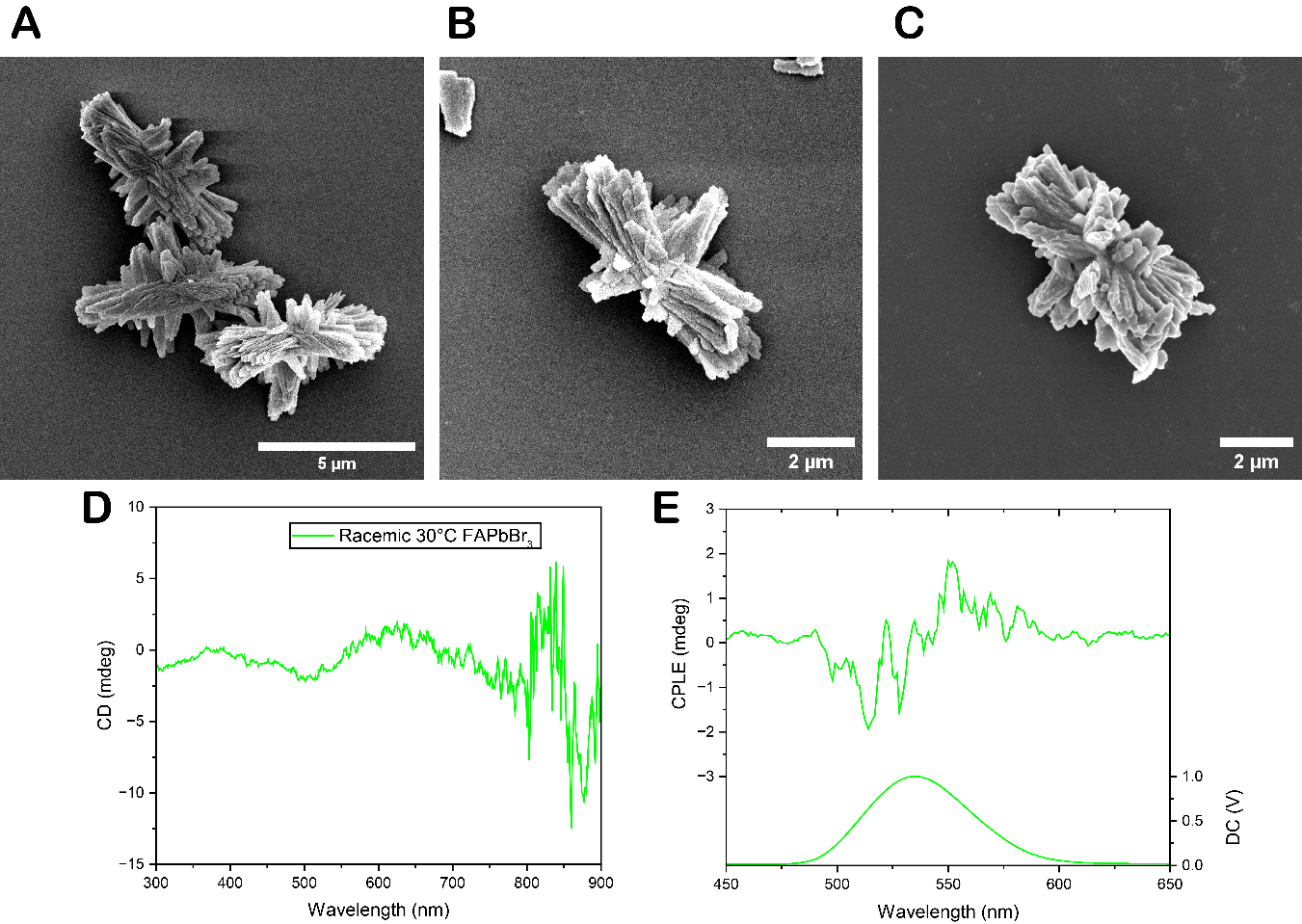
**

**Figure S10.** SEM, CD, CPLE data of racemic 30^°^C CHIPs, also known as ‘kayak’ particles. SEM images of a) Racemic CHIPs, b) racemic S-CHIPs, c) racemic P-CHIPs coated with FAPbBr_3_. d) CD and e) CPLE spectra of racemic P-CHIPs coated with FAPbBr_3_, which are near-zero.

**References**

[1] I. Levchuk, A. Osvet, X. Tang, M. Brandl, J. D. Perea, F. Hoegl, G. J. Matt, R. Hock, M. Batentschuk, C. J. Brabec, *Nano Lett.* **2017**, *17*, 2765.
